# Ribosome assembly factor Adenylate Kinase 6 maintains cell proliferation and cell size homeostasis during root growth

**DOI:** 10.1111/nph.16291

**Published:** 2019-12-02

**Authors:** Radka Slovak, Claudia Setzer, Mykola Roiuk, Jonas Bertels, Christian Göschl, Katharina Jandrasits, Gerrit T. S. Beemster, Wolfgang Busch

**Affiliations:** ^1^ Gregor Mendel Institute (GMI) Austrian Academy of Sciences Vienna Biocenter (VBC) Dr Bohr‐Gasse 3 1030 Vienna Austria; ^2^ Department of Plant Sciences University of Oxford South Parks Road Oxford OX1 3RB UK; ^3^ Max F. Perutz Laboratories (MFPL) Vienna Biocenter (VBC) Dr Bohr‐Gasse 9 1030 Vienna Austria; ^4^ Laboratory for Integrated Molecular Plant Physiology Research (IMPRES) Department of Biology University of Antwerp Groenenborgerlaan 171 2020 Antwerpen Belgium; ^5^ Plant Molecular and Cellular Biology Laboratory Salk Institute For Biological Studies 10010 N Torrey Pines Rd La Jolla CA 92037 USA

**Keywords:** *Arabidopsis thaliana*, cell elongation, cell proliferation, natural variation, ribosome biogenesis, root growth and development

## Abstract

- From the cellular perspective, organ growth is determined by production and growth of cells. Uncovering how these two processes are coordinated is essential for understanding organogenesis and regulation of organ growth.
- We utilized phenotypic and genetic variation of 252 natural accessions of *Arabidopsis thaliana* to conduct genome‐wide association studies (GWAS) for identifying genes underlying root growth variation; using a T‐DNA line candidate approach, we identified one gene involved in root growth control and characterized its function using microscopy, root growth kinematics, G2/M phase cell count, ploidy levels and ribosome polysome profiles.
- We identified a factor contributing to root growth control: *Arabidopsis Adenylate Kinase 6 (AAK6)*. *AAK6* is required for normal cell production and normal cell elongation, and its natural genetic variation is involved in determining root growth differences between Arabidopsis accessions. A lack of AAK6 reduces cell production in the *aak6* root apex, but this is partially compensated for by longer mature root cells. Thereby, *aak6* mutants exhibit compensatory cell enlargement, a phenomenon unexpected in roots. Moreover, *aak6* plants accumulate 80S ribosomes while the polysome profile remains unchanged, consistent with a phenotype of perturbed ribosome biogenesis.
- In conclusion, AAK6 impacts ribosome abundance, cell production and thereby root growth.

## Introduction

The growth of organisms depends on cell growth and cell division. These two processes need to be coordinated to maintain the characteristic sizes of cells (cell size homeostasis), organs and the whole organism. Two of the best‐characterized models of the coordination of cell division and growth are fission (*Schizosaccharomyces pombe*) and budding yeasts (*Saccharomyces cerevisiae*). There, cell size homeostasis is maintained by regulating cell division depending on growth. Blocking cell growth by nutrient deprivation leads to cell cycle arrest in G1 phase and cessation of cell division. Conversely, when cell division is blocked by arrest of cell cycle, at various stages, protein content continues increasing and cells keep growing until eventual cellular lysis (Mitchison & Creanor, 1971; Johnston *et al.*, 1977). This led to the hypothesis that cell division depends on cell growth that is monitored by a certain parameter, which has to reach a threshold value before cell division is initiated. Parameters proposed to control cell division initiation include a cell's age (Trucco, 1970), size (Fantes & Nurse, 1977; Johnston *et al.*, 1977) or biosynthetic capacity (the ability of a cell to synthesize organic molecules) (Unger & Hartwell, 1976; Brooks, 1977). The hypothesis of size‐dependent cell division is supported by the identification of the geometric size sensor Pom1 regulating cell division in fission yeast (Martin & Berthelot‐Grosjean, 2009; Moseley *et al.*, 2009). In budding yeast, methionyl‐tRNA concentration has been suggested as the decisive signal reporting cell's capacity to synthesize proteins (Unger & Hartwell, 1976). It is not surprising that protein synthesis is viewed as a key measure of cell growth (Polymenis & Aramayo, 2015) – proteins are needed for all cellular functions and represent the most abundant macromolecules in cellular dry mass. Interestingly, a significant fraction of eukaryotic proteome – 10–20% in yeast (Liebermeister *et al.*, 2014) and 7–16% in Arabidopsis accessions (Ishihara *et al.*, 2017) – is dedicated to assembly of ribosomes that in turn synthesize more proteins. It is the ribosome abundance and energy that are implicated to be the growth‐limiting factors in higher eukaryotes (slow‐growing respiring cells), in contrast to limitation of growth by gene transcription and/or translation in rapidly growing microorganisms (Kafri *et al.*, 2016).

However, there is a fine balance between the benefits of ribosomes and their costs. Specifically, the production and use of ribosomes are the most costly expenses of growth (Russell & Cook, 1995; Rolfe & Brown, 1997; Warner, 1999). The multi‐step, energy‐demanding ribosome biogenesis includes synthesis of pre‐ribosomal RNAs in the nucleolus, synthesis of ribosomal proteins in the cytoplasm, pre‐ribosome assembly in the nucleolus, processing of pre‐ribosomal RNAs and transport of ribosome subunits to their final workplace in the cytoplasm (excellently characterized in yeast; Woolford & Baserga, 2013).

Only a fraction of yeast ribosome biogenesis orthologues in plants have been functionally characterized (Barakat *et al.*, 2001; Weis *et al.*, 2015). At the same time, mutant phenotypes of genes involved, or with a suggested role, in ribosome biogenesis (*toz*,* swa1*,* nuc‐l1/paralell1*,* ebp1*) (Shi *et al.*, 2005; Horváth *et al.*, 2006; Griffith *et al.*, 2007; Kojima *et al.*, 2007; Petricka & Nelson, 2007) phenocopy ribosomal structural protein defects. The developmental impact of proteins involved structurally or functionally in ribosome biogenesis overlaps. The respective mutant phenotypes generally occur in tissues requiring high protein synthesis and/or transcript of a critical gene involved in promoting proliferation and development (reviewed by Byrne, 2009).

Mutations in a few Arabidopsis orthologues of yeast ribosomal assembly or structural proteins have been associated with a phenomenon termed compensatory cell enlargement (CCE) (Fujikura *et al.*, 2009). CCE is an excessive postmitotic cellular growth that follows a decrease of cell production that is thereby compensated for at the organ level. CCE occurs in plants and animals as well (Hemerly *et al.*, 1995; Neufeld *et al.*, 1998; De Veylder *et al.*, 2001; Ferjani *et al.*, 2007; Roeder *et al.*, 2010; Worley *et al.*, 2013). In plants, CCE has been described in organs of determinate growth (e.g. leaves (Ferjani *et al.*, 2007), petals (Randall *et al.*, 2015) and sepals (Roeder *et al.*, 2010)). However, for organs of indeterminate growth – such as roots – it has been thought not to occur (Ferjani *et al.*, 2007).

Shoots and roots grow throughout the entire lifetime of higher plants. This growth is enabled by cell divisions and postmitotic cellular expansion specifically at the shoot and root apices. In the root apex, cell divisions occur in the meristematic zone directly adjacent to the quiescent centre and the stem cell niche, while the postmitotic growth starts when cells exit the meristematic zone. The postmitotic increase in size of root cells is accompanied by endoreplication (Hayashi *et al.*, 2013). According to the current understanding, the coordination of cell division and cell growth in the root apex is flexibly coupled (Yang *et al.*, 2017). Moreover, phytohormone crosstalk (e.g. auxins‐cytokinins, auxins‐brassinosteroids and gibberellins) determines the root apex zonation noncell‐autonomously (Ubeda‐Tomás *et al.*, 2009; Moubayidin *et al.*, 2010, Ubeda‐Tomás *et al.*, 2012, Chaiwanon & Wang, 2015). Much of the work on growth regulation in plants has focused on environmental stresses (Guan *et al.*, 2013; Wang *et al.*, 2014), hormones (Ishida *et al.*, 2009; Moubayidin *et al.*, 2010) and cell cycle genes (De Schutter *et al.*, 2007; Inagaki & Umeda, 2011). Despite indications of a role for ribosome biogenesis in plant development and growth, its involvement in the coordination of cell production and cell expansion in the root development remained unknown.

Here we report on the identification of a factor contributing to the control of root growth – the *Arabidopsis Adenylate Kinase 6 *(*AAK6*) that affects cellular growth by impacting the assembly of ribosomes. We show that 80S ribosome complexes overaccumulate in mutants of this gene (*aak6*), orthologue of yeast ribosome assembly factor Fap7. Cell production is lower in *aak6* plants, resulting in fewer cells in *aak6* roots. This reduced cell production is partially compensated for by additional postmitotic cellular growth. Nevertheless, *aak6* roots are overall shorter and slower‐growing. Cyclin expression and ploidy levels in the *aak6* mutant suggest that AAK6 activity is required for normal cell cycle progression during cell proliferation and endoreplication. Moreover, association of natural allelic variation of *AAK6* with measured root growth differences suggest a contribution of ribosome assembly to root organ growth among natural Arabidopsis accessions. This work thereby demonstrates a link between ribosome assembly and growth rate modulation.

## Materials and Methods

### Plant material

We used seeds of 252 *Arabidopsis thaliana* accessions (Supporting Information Table S1; Notes S1) in the genome‐wide association study (GWAS).

Available T‐DNA insertion lines within 4000 bp upstream or downstream (Table S2) for two of the top five associated single nucleotide polymorphisms (SNPs) (Fig. S1d) were ordered from Nottingham Arabidopsis Stock Centre, for candidate gene prioritization (Notes S2).

Seed size variance was controlled by two sieving steps using Retsch sieves (60.131.000250 and 60.131.000280, Retsch GmbH, Haan, Germany), for all genotypes. Seeds < 250 µm and > 280 µm in size were excluded.

### Growth conditions

Seeds were surface‐sterilized, plated, stratified and cultured as described in Slovak *et al.* (2014). In particular, after stratification, seeds were transferred to the growth chamber and allotted 48 h for germination. At this time point, on the second day after stratification (DAS2), the vast majority of the seedlings germinated.

### Growth quantification

Plates with growing seedlings were moved to the image acquisition room once a day, from DAS3 (corresponding to the first day after germination) for 5 d until DAS7, scanned and, after imaging, returned immediately to the growth chamber. The root traits were quantified by high‐throughput plugin BRAT including manual quality control step, as described in Slovak *et al.* (2014).

### Genome‐wide association studies

We evaluated 78 GWASs executed on 14 root morphology and two growth rate traits evaluated for five time points of a 5‐d‐long growth assay, four end points respectively for the ‘rate' traits. The GWASs are considered significant when they pass the 5% Benjamini–Hochberg–Yekutieli false discovery rate (FDR) threshold. Arithmetic means (*n* > 5) of 252 accessions were utilized for these GWASs. The computations were performed on a Gregor Mendel Institute computer cluster using algorithms described in Seren *et al.* (2012). We took into account SNPs with minor allele counts ≥ 12.

#### Effects of seed size restriction on GWASs

We compared counts of significant genome‐wide associations between the current size‐controlled dataset and our previous study that did not control seed size (Slovak *et al.*, 2014). Both datasets were downsized to 160 accessions (representing the overlap of both studies). We compared cumulative counts from across 78 GWASs executed on 14 root morphology and two growth rate traits evaluated for five time points of a 5‐d‐long growth assay, four end points respectively for the ‘rate' traits.

### Plasmid construction and plant transformation

The following plasmids were constructed by In‐Fusion cloning (Clontech Laboratories Inc., Mountain View, CA, USA) into the modified pGreen0229 that has *p35S:PM‐mCherry* reporter gene for visual selection of positive transformants (Hellens *et al.*, 2000; Emami *et al.*, 2013; Satbhai *et al.*, 2017), kindly provided by our colleague Dr Santosh Satbhai. The following plasmids were verified by Sanger sequencing and the respective T–DNAs transferred by *Agrobacterium*‐mediated transformation by floral dipping (Weigel & Glazebrook, 2006). We used a Zeiss Discovery.V8 stereo‐microscope to visually select transgenic seeds, and finally obtained homozygous transgenic seeds in the third generation. The primers are listed in Table S3.

#### *35S:AAK6* overexpressor line

The *AAK6* coding sequence, amplified from Columbia‐0 (Col‐0) genomic DNA (primer pair P18, P19; Table S3), was inserted in the modified pGreen0229 under the control of the *35S promoter* and the translational enhancer (*TL‐TEV*) (primer pair P16, P17; Table S3) to form an overexpression vector.

The *35S:AAK6* overexpression was validated (see Methods S1; Fig. S2a).

#### Complementation of *aak6* by *AAK6* natural alleles

The DNA fragment, amplified from genomic DNA of Bå4‐1, Col‐0 and Tha‐1 accessions, respectively (corresponding to the 1823 bp region in the reference genome of Col‐0 – covering 788 bp upstream to 241 bp downstream from CDS), was inserted into the modified pGreen0229 (primer pair P20, P21; Table S3).

We generated 50 independent insertion lines for each of the three natural *AAK6* alleles, checked the segregation ratio of the mCherry marker in the T_2_ generation and chose seven independent lines, with segregation ratio closest to 3 : 1, for further propagation. All inserted *AAK6* alleles complemented the dwarf phenotype of the original *aak6* background. In the third generation, we obtained seven independent, homozygous insertion lines for each of the three *AAK6* alleles (from Bå4‐1, Col‐0 and Tha‐1 accessions).

#### *pAAK6:AAK6‐Clover* reporter line

The expression vector was constructed using Multisite Three‐fragment Gateway cloning (Thermo Fisher Scientific, Vienna, Austria). The native promotor (788 bp upstream of the translation start site) and the *AAK6* coding sequence without the stop codon were cloned into Gateway entry vectors pDONR P4‐P1r and pDONR221, respectively (primer pairs P22 and P23, and P24 and P25; Table S3). The Clover reporter transgene in pENTR P2r‐P3 was kindly provided by Dr Tomokazu Kawashima. These three entry vector clones and the destination vector pK7m34GW were then recombined in the Multisite Gateway LR+ recombination reaction to create the *pAAK6:AAK6‐Clover* expression vector. This plasmid was verified by Sanger sequencing and the respective T‐DNA transferred into the Col‐0 background using *Agrobacterium* transformation (Weigel & Glazebrook, 2006). Positive transformants were selected on kanamycin 1 × MS medium agar plates, plants were propagated on soil and second‐generation seedlings were used for analysis of the spatial pattern of AAK6‐Clover protein expression *in vivo*.

### Root anatomy measurements

#### Static measurements of cell lengths and root zones by confocal microscopy

Wild‐type Col‐0 and *aak6* mutant (SALK_015289) seeds were surface‐sterilized, stratified and cultured on 1× MS, 1% sucrose, 0.8% agar plates and seedlings were grown until DAS5. Seedlings were incubated for 10 s with 15 μg ml^–1^ propidium iodide, washed, transferred to standard microscopy slides, and mounted in MonoQ water (VBCF, Vienna, Austria) under coverslip. An LSM 700 Axio Observer.Z1 confocal microscope (Zeiss) with a motorized stage was used for confocal image acquisition. Using zen2010 software (Zeiss), ×20 dry objective (Zeiss Plan‐Apochromat ×20/0.8) we acquired 12 z‐stacks of > 20 planes spaced at 2.4 µm (400 × 400 μm) for each root, with a resolution of 0.78 μm per pixel. Propidium iodide was excited at 488 nm with 10% laser power and detected at 576–700 nm. Twelve stacks were stitched for each root using the fiji stitching plugin (Preibisch *et al.*, 2009) post‐acquisition.

We measured root cortex cell lengths using the manual segmentation tool in fiji starting with the first cell after the endodermis/cortex initial and continuing cell‐by‐cell past the maturation zone boundary. The first cell being twice as long as the previous one, with a longer subsequent cell (to skip dividing cells) defined the rapid elongation zone start. The meristem zone length was measured from the root quiescent centre to the rapid‐elongation‐zone boundary using the zone determination as described earlier. The number of cells in the meristematic zone was determined by counting the cells after the endodermis/cortex initial until the first cell of the rapid elongation zone. The mature zone start is defined as a position after which cell elongation stops and is detected in the longitudinal dimension after the rapid elongation zone where a cell has approximately the same length as the following one. The mature cell length was calculated as the average of first five cells in the mature zone.

We evaluated these measurements for both right and left cortex cell files in the root middle plane and subsequently calculated the average for each seedling. We analysed 10 seedlings for wild‐type Col‐0 and *aak6* genotypes each, in blinded analyses.

#### Kinematic analyses of root zones, cell production and postmitotic growth

Wild‐type Col‐0 and *aak6* mutant (SALK_015289) seeds were surface‐sterilized (with 70% ethanol for 1 min and 5% bleach for 5 min and rinsed five times with MilliQ water) and plated on 1 × MS, 0.1% sucrose, 0.8% agar plates. Plates were stored for 3 d at 4°C, after which they were placed vertically in a growth chamber under constant conditions (16 h : 8 h, light : dark, 20°C, 74.5 μmol PAR m^−^^2^ s^−1^).

Kinematic analysis of cell division and elongation was performed on DAS8 (5–6 d after germination for the majority of plants). To obtain velocity measurements, black‐toner particles (Hewlett‐Packard, Belgium BVBA, Diegem, Belgium) were applied on the roots and their displacement measured after 1 h. Images of the particle‐sprinkled roots at start and end time points were acquired directly from the plates using a binocular microscope SMZ1000 (Nikon) at ×8 magnification equipped with a digital camera (AxioCam ICc 1; Zeiss), connected to a PC running axiovision v.4.8.2 software (Zeiss). Roots were then whole‐mounted on microscope slides in 1 × MS liquid medium, 0.1% sucrose and cortical cells visualized by an Axio Scope.A1 microscope (Zeiss) using differential interference contrast (DIC) optics at ×20 magnification. Images were again captured with an AxioCam ICm 1 camera (Zeiss) controlled by axiovision v.4.8.2 software (Zeiss).

Cell division and elongation parameters were determined by kinematic calculations as described previously (Beemster & Baskin, 1998) with minor modifications. In particular, the cell production was calculated by dividing the final velocity by the mature cell length. The cortical cell lengths were measured in imagej (Schneider *et al.*, 2012). A ruler image was used for calibration. The cell flux (h^−1^) was calculated by dividing the velocity (µm h^−1^) by the cell length (µm) at the respective discrete distance points. The meristematic zone length was determined as the distance between the quiescent centre and the first point where the flux became ≥ 95% of the average of the remaining flux data points in the growth zone (i.e. when the flux curve reaches the plateau). The elongation zone length was determined as the distance between the meristematic zone boundary and the first point where the velocity became ≥ 95% of the average of the remaining velocity data points in the growth zone (i.e. when the fitted velocity curve reaches the plateau).

### Cell proliferation analysis

To count root meristematic zone cells in the G2/M phase, we introgressed the *pCycB1;1:CycB1;1DB‐GFP* reporter generated by P. Doerner (Ubeda‐Tomás *et al.*, 2009) into Col‐0 and *aak6* –/– backgrounds, respectively. We phenotyped the F_3_ generation homozygous for *CycB1;1DB‐GFP* (+/+) in Col‐0 and *aak6* –/– homozygous backgrounds, respectively, on DAS5.

Before imaging, seedlings were stained 10 s by propidium iodide (15 μg ml^–1^), washed in MonoQ water and mounted on microscope slides in MonoQ water (VBCF, Vienna, Austria) under a coverslip. Z‐stacks were acquired using a confocal microscope LSM 700 Axio Observer Z1 (Zeiss) with PlanApochromat ×20/0.8 objective, controlled by zen2010 software (Zeiss). Z‐stacks covered the range of planes from the root surface past the root middle plane with a 2.4 µm interval. In *XY* dimension, the stack comprised longitudinally the root meristematic and part of the rapid elongation zone within a single tile (400 μm^2^). A 488 nm laser, at 10% power, was used for green fluorescent protein (GFP) and propidium iodide excitation. Emitted light was recorded in a single track separated into two channels (shortpass 555 nm; longpass 560 nm; dichroic beam splitter (DBS) 572 nm).

Using the z‐stack confocal images described earlier, we first established the position of the root middle plain and manually registered and counted CycB1;1DB‐GFP‐positive cells in the root meristematic zone in all cell files from the surface to the root middle plain (disregarding columella and lateral root cap) using the fiji roi manager tool. We scored 10 seedlings of wild‐type Col‐0 (*CycB1;1DB‐GFP +/+*) and *aak6* −/− (*CycB1;1DB‐GFP +/+*) genotypes each, in a blinded analysis.

### Flow‐cytometric ploidy level assessment

Five‐millimetre‐long root tips were harvested from *c.* 100 seedlings, on DAS5, and placed in 250 ml of cold nuclei Extraction Buffer (Sysmex Partec GmbH, Görlitz, Germany) in a Petri dish on ice. Root tips were chopped with a new razor blade exchanged between every sample. The nuclei suspension was incubated for 2 h in 1 ml of CyStain PI Absolute P (Sysmex Partec GmbH) staining buffer in the dark on ice. The stained homogenate was filtered through a 30 μm CellTrics filters (Sysmex Partec GmbH). Stained particles were excited with 561 nm (110 mW) and 488 nm (100 mW) lasers and emission was detected for propidium iodide‐stained particles (610/20 nm) and forward scattering (488/10 nm). Fluorescence intensity was recorded for 10 000 particles for each sample. facs diva software (BD Lifesciences, Franklin Lakes, NJ, USA) was used for gating the nuclear populations. We analysed six pooled biological samples each for the Col‐0 wild‐type and the *aak6* mutant.

### Ribosome/polysome profiles

Plants were grown on 1 × MS agar plates for 3 wk after germination. Fresh polysome extraction buffer was prepared as follows: 0.2 M Tris‐HCl (pH 9.8), 0.2 M KCl, 0.025 M EGTA, 0.035 M MgCl_2_, 1% Detergent mix (1% (w/v) Brij35, 1% Triton X‐100, 1% (v/v) Igepal CA 630, 1% Tween 20), 1% sodium deoxycholate, 1% polyoxyethylene 10 tridecyl ether, 5 mM dithiothreitol, 1 mM phenylmethylsulfonyl fluoride, 50 μg ml^–1^ cycloheximide, 50 μg ml^–1^ chloramphenicol and 0.5 mg ml^–1^ heparin. Whole seedlings were harvested and manually grounded in liquid nitrogen. A unit of weight (1 g) of tissue powder was incubated for 10 min with 2 units of volume (ml) of polysome extraction buffer, on ice. Samples were centrifuged at 16 000 ***g*** for 15 min at 4°C, in an Eppendorf microcentrifuge. Supernatant was poured through Miracloth (Merck, Darmstadt, Germany) into a new chilled tube. Centrifugation at 16 000 ***g*** for 15 min was repeated at 4°C. 12 ml of 5–45% (w/v) linear sucrose gradients were prepared in 0.04 M Tris, 0.02 M KCl and 0.01 M MgCl_2_ salt solution (with 50 μg ml^–1^ cycloheximide, 50 μg ml^–1^ chloramphenicol) in thick‐walled polycarbonate tubes using Gradient Master 107 (Biocomp Instruments, Fredericton, Canada). Clarified ribosome/polysome extracts (500 μl) were layered on top of sucrose gradients. Tubes with samples and sucrose gradients were centrifuged at 40 000 ***g*** for 2.5 h, at 4°C, in an SW40 rotor, Optima L‐70 ultracentrifuge (Beckman Coulter, Brea, CA, USA). UV 254 nm absorbance was measured using an ÄKTAFPLC machine (GE Healthcare Life Sciences, Vienna, Austria). We analysed three biological pooled replicates each originating from 144 plants for the wild‐type Col‐0 and *aak6* mutant genotype.

## Results

### A seed size‐controlled root trait dataset identifies an unknown root growth factor

Previous studies on root growth and gravitropism suggested that early root traits are impacted by seed size and other maternal effects (Elwell *et al.*, 2011). Additionally, we observed a correlation between average seed size and average root growth traits (published previously for 163 accessions) (e.g. for total length_DAS7_, *r* = 0.43; Fig. S1a; Table S4; Methods S2). To minimize maternal and seed size effects of 252 accessions, we used sieves to restrict seed sizes to 250–280 μm. We then quantified 16 root growth traits over 5 d, from DAS3 to DAS7, using the BRAT phenotyping (Slovak *et al.*, 2014). While these new seed size‐controlled early root growth traits correlate with the traits from the dataset not controlling seed size (Slovak *et al.*, 2014) (e.g. root length_DAS7_, Pearson's *r* = 0.75; Table S4), restricting seed size variance led to a decrease in variance within accessions (median accession's SD_DAS7_ = 2.6 mm → median accession's SD_DAS7_ = 1.8 mm) as well as between accessions (e.g. for primary root length_DAS7_, SD_DAS7_ = 2.9 mm → SD_DAS7_ = 2.3 mm) (Table S4). Despite the variance reduction in early root growth traits, the remarkable natural variation of root traits was still preserved (e.g. *c.* three‐fold difference in primary root growth on DAS7 between accessions: from 1.6 to 4.6 mm d^–1^; Fig. S1b). Upon restriction of seed size variance, broad‐sense heritability increased for most traits (e.g. primary root length_DAS7_, *H* = 0.49 → *H* = 0.50; average root growth rate: *H* = 0.45 *→* *H* = 0.50; Table S4). Using our seed size‐controlled phenotype dataset, we then conducted GWASs using the EMMA(X) method that corrects for population structure (Kang *et al.*, 2008, 2010; Seren *et al.*, 2012). We focused on dissecting the molecular basis of the quantitative root growth, despite having conducted 77 more GWASs for additional root growth traits (Table S5, Spreadsheet 1). Although no genome‐wide association crossed the 5% FDR significance threshold for the root growth rate on DAS7, we identified multiple distinct genome‐wide association peaks (Fig. S1c).

**Figure 1:**
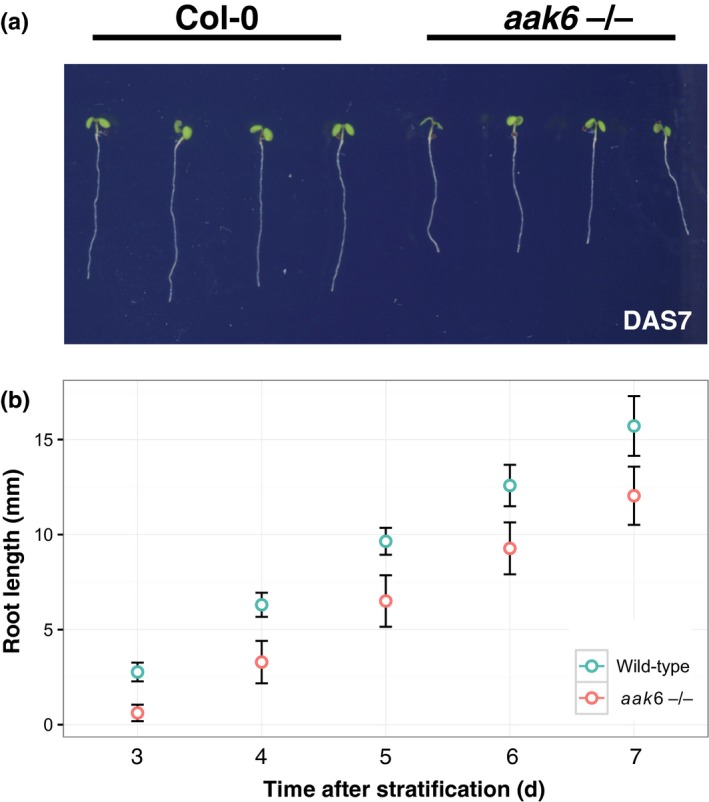
Primary root growth of *Arabidopsis thaliana* 1 wk after stratification. (a) Representative seedlings of wild‐type and *aak6* genotype, on day 7 after stratification (DAS7). (b) Primary root length on DAS3–DAS7; *t*‐test, *P* < 0.001 on each of the 5 d, means ± SD.

**Figure 2:**
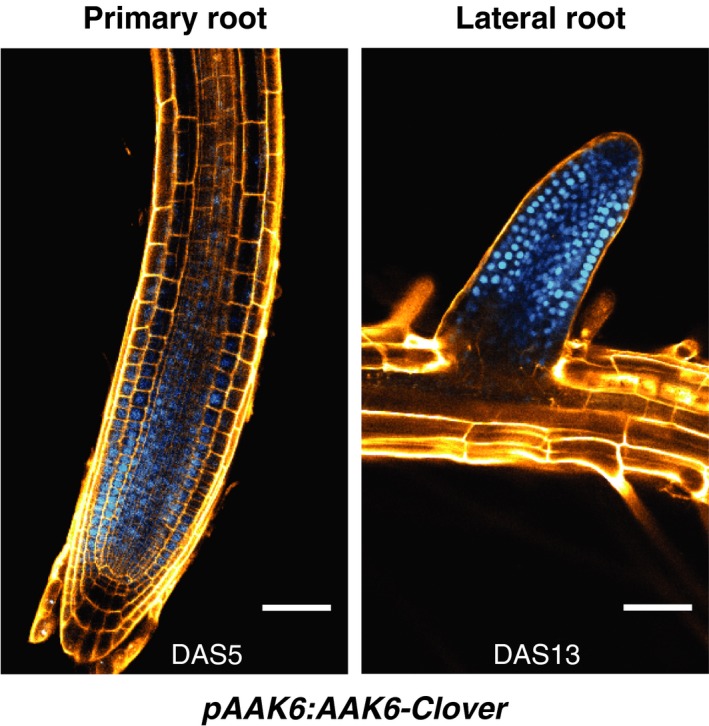
Expression of *pAAK6:AAK6‐Clover* translational reporter. AAK6‐Clover translational fusion protein (shown in blue) has nuclear localization in files of all cell types in primary and lateral root meristems of *Arabidopsis thaliana* (on 5^th^ and 13^th^ day after stratification, DAS5 and DAS13). Cell walls are stained by propidium iodide (shown in orange). Bars, 50 μm.

We focused on two of the top five associated genomic regions (Fig. S1d). Because the top SNP, identified by GWAS, frequently is not the causal SNP, but rather only marks the genomic region in which causal nucleotide polymorphisms reside, we screened the available T‐DNA lines targeting genes within 8 kb regions comprising the top associated marker SNPs (Table S2). One of these lines (SALK_015289) developed significantly shorter roots (*P* < 0.001; Fig. 1). The data therefore suggested that this line is defective in a gene required for root growth. SALK_015289 has a T‐DNA insertion in the 5′ untranslated region of *Arabidopsis Adenylate Kinase 6* (*AAK6*; AT5G60340) leading to severe downregulation of *AAK6* expression level (Fig. S2a). Importantly, using the Col‐0 genomic region (1823 bp, 788 bp upstream, 241 bp downstream) we could complement the known *aak6* dwarf mutant phenotype (Feng *et al.*, 2012) (Fig. S3) and partially complement the shorter root and slower growth (Fig. S4) excluding a background mutation causing these phenotypes. Consistent with a requirement in normal root growth, *pAAK6:AAK6‐Clover* reporter construct is expressed in nuclei of all cell types in primary and lateral root meristematic zones (Fig. 2). Furthermore, *AAK6* shows general predominant expression in actively dividing tissues in Arabidopsis (TraVa RNAseq expression atlas; Klepikova *et al.*, 2016). Nonetheless, overexpression of *AAK6* under *35S* promoter did not lead to longer roots or their faster growth (Fig. S2b). In conclusion, the data show that the *AAK6* gene is required for normal root growth but its overexpression is not sufficient for faster root growth.

### AAK6 natural allelic variation is involved in root growth rate determination

**Figure 3:**
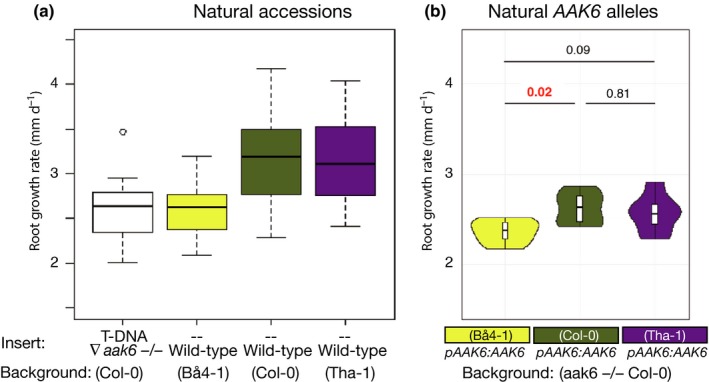
Complementation of *Arabidopsis thaliana aak6* mutant with natural *AAK6* alleles. (a) Root growth rate of natural accessions and *aak6* mutant on day 7 after stratification (*n* > 15). Whiskers represent the last value at a distance smaller than 1.5 × the interquartile range (IQR). (b) Root growth rate of transgenic plants carrying natural *AAK6* DNA fragment in *aak6 *−/− background on day 7 after stratification. Shown are medians with interquartile range (black line in white box) and density distribution in a violin plot trimmed to the range of the data (*n* = 7 independent insertion lines for each of the three constructs); Tukey honestly significant difference adjusted *P*‐values are indicated above the violin plots.

The GWAS, followed by the mutant phenotype, suggested that *AAK6* natural genetic variation matters in determining the different root growth rates observed in our panel of accessions. However, to directly test this, we used a transgenic complementation approach. We classified the accessions into haplotypes and tested whether transforming the slow‐growing *aak6* mutant with a genomic fragment – including *AAK6* promotor and coding region – from one of the slower‐growing haplotypes (Bå4‐1) would result in plants displaying slower root growth than plants transformed with an allele from the faster‐growing haplotypes, such as Col‐0 or Tha‐1. We generated 50 independent complementation lines for each of the three alleles, and chose seven for each allele – with the mCherry selection marker segregation ratio closest to 3 : 1 in the T_2_ generation. We then phenotyped the T_3_ generation homozygous seedlings. Complementation of the loss‐of‐function *aak6* mutant with the allele from the slower‐growing haplotype resulted in significantly slower root growth of seedlings than observed in plants complemented with alleles from the faster‐growing haplotypes (Tukey honestly significant difference, *P*_adj_ = 0.02 and *P*_adj_ = 0.09 for the Col‐0 and Tha‐1 genomic fragment, respectively; jointly tested allele effect via ANOVA, *P* = 0.02; Fig. 3). As there are four nonsynonymous amino acid substitutions in the coding region of *AAK6* genomic fragments used for *aak6* complementation (Table S6), these are the prime candidates for causing the phenotypes. In summary, we show that allelic variation of the tested *AAK6* genomic fragments determines root growth differences and therefore conclude that natural genetic variation in the *AAK6* gene is involved in determining the root growth differences between natural accessions.

### AAK6 is required for normal cell production and normal cell elongation

To understand the role of AAK6 in root development, we examined plants homozygous for *aak6*. These have shorter roots and decreased root growth rate. Therefore, cell proliferation and/or cell elongation could be altered as these are the two main processes that determine the root growth rate. We assessed this by two independent sets of experiments (confocal microscopy and kinematic analysis).

**Figure 4:**
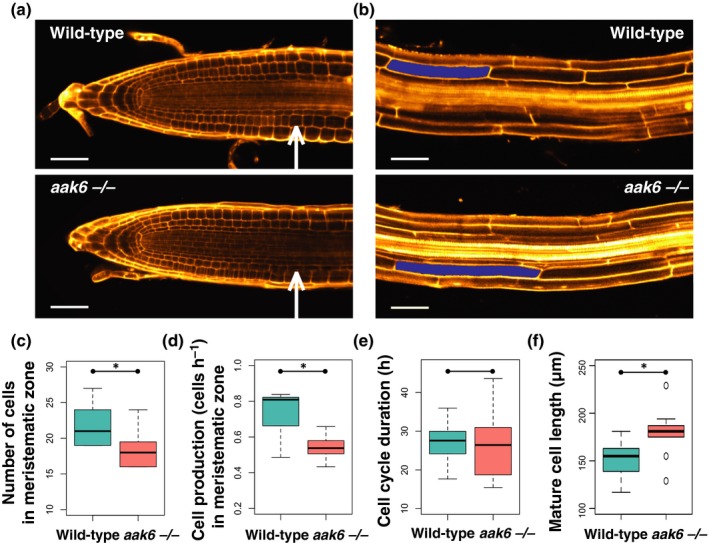
Effect of lack of AAK6 on root anatomy, cell production and elongation in *Arabidopsis thaliana*. (a) Wild‐type and *aak6 *−/− root meristematic zone. Cell walls are stained by propidium iodide (shown in orange). The white arrows indicate the first cortex cell in the zone of rapid elongation marking the end of the meristematic zone on day 5 after stratification (DAS5). Bars, 50 μm. (b) Image section from wild‐type and *aak6 *−/− root maturation zone on DAS5. Representative cortical cells are highlighted in blue. Cell walls are stained by propidium iodide (shown in orange). Bars, 50 μm. (c) Meristematic zone cell numbers in wild‐type and *aak6 *−/− roots evaluated in the cortex cell file on confocal microscopy images on DAS5. Whiskers represent the last value at a distance smaller than 1.5 × the interquartile range (IQR) (*t*‐test: *, *P* < 0.05). (d) Meristematic zone cell production (cells h^–1^), determined by kinematic analysis, on DAS8; whiskers < 1.5 × IQR (*t*‐test: *, *P* < 0.05). (e) Cell cycle duration (h), determined by kinematic analysis, on DAS8; whiskers < 1.5 × IQR (*P* = 0.88). (f) Wild‐type and *aak6 *−/− mature cortical cell length in the root, evaluated on confocal microscopy images, on DAS5; whiskers < 1.5 × IQR (*t*‐test: *, *P* < 0.05).

First, we investigated cell proliferation. Using confocal microscopy, we observed significantly fewer cells (*P* = 0.03) in the meristematic zone of *aak6* roots (Figs 4a,c, S5a,b), although the meristematic zone length was unaffected (*P* = 0.97). Kinematic analyses of root apical growth revealed that the *aak6* root meristematic zone produces fewer cells compared with wild‐type plants (*P* = 0.01; Fig. 4d). Consistent with the confocal microscopy‐based observations, we independently observed a lower number of meristematic cells in the *aak6* mutant using DIC microscopy (*P* = 0.04). Furthermore, the length of the meristematic zone again did not differ between the *aak6* and the wild‐type (Fig. S5c,d). The average cell cycle duration, determined by kinematic analysis, is similar in the *aak6* mutant and the wild‐type (Fig. 4e). We therefore conclude that the lower number of the proliferating cells in the *aak6* meristematic zone is the predominant reason for the significantly lower cell production in the *aak6* roots.

Next, we investigated the involvement of cell elongation in the decreased growth of the *aak6* roots. We measured cell lengths along the longitudinal axis of the root (Fig. S5). While the length of cells leaving the meristematic zone was not significantly different between *aak6* and the wild‐type, the final mature cell length of the *aak6* mutants was, contrary to our expectations, 19% longer in the slower‐growing roots of the *aak6* mutant (cortex cell tier, confocal microscopy, *P* < 0.01 (Fig. 4b,f); kinematic analyses, *P*‐value < 0.01 (Fig. S5d)). The *aak6* mature cortical cells are generally affected – the whole mature cell size distribution is shifted towards longer cells (Fig. 4f). This could be the result of faster local cell elongation rates and/or the longer time a cell spends transiting the elongation zone (average duration of cell elongation). However, the profile of local relative cell elongation rates was not significantly different between *aak6* mutant and wild‐type roots (kinematic analysis of the average local cell elongation rate, *P* = 0.93; Fig. S6a,b) and we observed only a statistically insignificant increase in the average time a cell spends in the elongation zone (9 h for wild‐type cells and 13 h for *aak6* cells, *P* = 0.50; Fig. S6c). Given the exponential nature of the elongation process, a small increase in the duration of the postmitotic cell elongation has a strong effect on the final mature cell size and eventually leads to significantly larger mature cells.

Taken together, we observed a reduced cell production and an increased cell growth in the *aak6* root. AAK6 activity is thus required for normal cell production and normal cell elongation. Moreover, given that the AAK6‐Clover translational fusion protein was expressed in nuclei of cells in the primary and lateral root meristematic zones (Fig. 2), we conclude that the AAK6 effect on the root cell expansion in the elongation zone is rather indirect. Last but not least, the *aak6* mutation uncovered an unexpected case of a phenomenon termed the compensatory cell enlargement – in an organ of indeterminate growth – the Arabidopsis root.

### AAK6 is required for normal cell proliferation while lack of AAK6 enhances endoreplication

**Figure 5:**
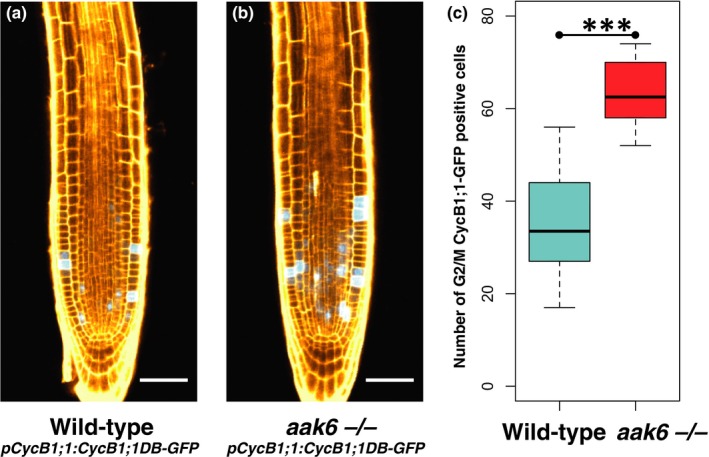
*Arabidopsis thaliana aak6 *−/− root meristematic zone contains more G2/M‐positive cells. (a, b) Representative confocal images showing the expression pattern of the *pCycB1;1:CycB1;1DB‐GFP* marker (shown in cyan), on day 5 after stratification in median longitudinal optical sections of wild‐type (a) and *aak6 *−/− root apices (b). Cell walls are stained by propidium iodide (shown in orange). Bars, 50 μm. (c) Box plot of G2/M CycB1;1‐GFP‐positive cells in wild‐type and *aak6 *−/− root apices; whiskers represent minimum and maximum values (*t*‐test: ***, *P* < 0.001).

The kinematic analysis of the cellular dynamics suggested that average cell cycle duration is similar in the *aak6* mutant and the wild‐type. We therefore independently assessed if the duration of cell cycle phases is altered in *aak6* roots. For this, we introgressed the G2/M phase reporter (*pCycB1;1:CycB1;1DB‐GFP*) into *aak6* and wild‐type plants. Interestingly, the *aak6* mutant showed significantly more CycB1;1‐GFP‐positive cells than did the wild‐type (*P* < 0.001; Fig. 5). The higher number of cells observed in the G2/M phase suggests an increase of G2/M phase duration. Longer G2/M despite the same cell cycle duration in turn suggests that G1/S is shorter.

**Figure 6:**
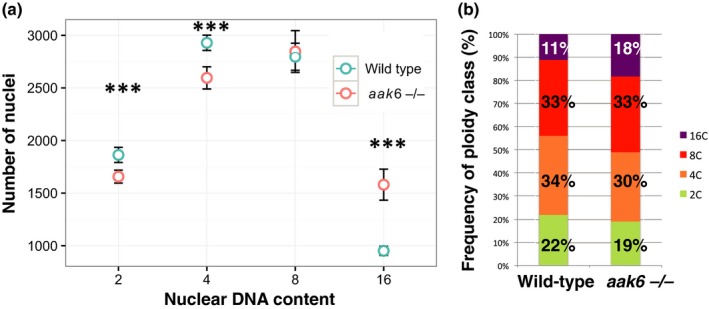
Nuclear ploidy levels in *Arabidopsis thaliana* wild‐type and *aak6* root apices. For each of the two genotypes, we collected six pooled 5 mm root tip samples on day 5 after stratification. 10 000 Arabidopsis nuclei were analysed from each sample. (a) Flow‐cytometry profile (means ± SD) (*t*‐test: ***, *P* < 0.001). (b) Frequency of nuclear ploidy classes.

As the endocycle only contains the G1/S transition, accelerated G1/S would lead to faster endocycling. We therefore set out to assess the extent of cell cycling and endocycling in the root tip cells. The nuclear ploidy levels were measured by flow‐cytometric analysis of propidium iodide‐stained nuclei isolated from 5‐mm‐long root tips. Interestingly, *aak6* roots have significantly lower proportions of 2C and 4C nuclei and a significantly increased proportion of 16C nuclei (*t*‐test, *P* < 0.001; Fig. 6; Table S7). Therefore these data do indeed demonstrate increased endoreplication in the *aak6* mutant root (for further discussion, see Notes S3).

Overall, AAK6 is required for normal cell production in the root meristematic zone and normal progression through the cell cycle. A lack of AAK6 significantly lowers cell production and alters cell cycle progression, leading to increased endoreplication and CCE.

### A lack of AAK6 leads to accumulation of 80S ribosomes

**Figure 7:**
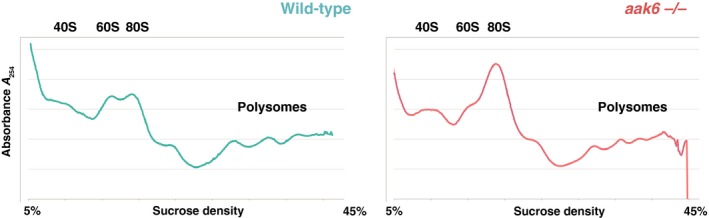
Ribosome/polysome profiles. Spectrophotometric profiles of UV absorbance (254 nm) of sugar gradients. Ribosome/polysome extracts were obtained from 3‐wk‐old whole seedlings of *Arabidopsis thaliana*. Ribosomal particles were fractionated through a 5–45% (w/v) sucrose gradient by 2.5‐h‐long ultracentrifugation.

While previous work has demonstrated an *in vitro* adenylate kinase activity of AAK6, its functional role remained unclear (Feng *et al.*, 2012). Interestingly, yeast (Fap7) and human (hCINAP) homologues of AAK6 are implicated in small ribosomal subunit maturation (Strunk *et al.*, 2012; Bai *et al.*, 2016). To test the hypothesis of AAK6 involvement in ribosome biogenesis, we evaluated ribosome‐polysome profiles from wild‐type and *aak6* mutant plants. The respective cell lysates were fractionated by centrifugation in 5–45% sucrose gradient to examine the relative abundance of ribosomal subunits, ribosomes and polysomes. Compared with the wild‐type, *aak6* seedlings contained increased amounts of 80S ribosomes (Figs 7, S7). These data suggest that upon AAK6 deficiency, the ribosome biogenesis becomes stalled at the 80S maturation step. Overall, our results show that an absence of AAK6 results in accumulation of 80S ribosomes in Arabidopsis, thereby linking ribosome biogenesis, cell production and postmitotic cellular growth in determining root growth.

## Discussion

Despite the essentiality of ribosomes for growth and development, it was not known how modulating ribosome abundance impacts cell production and cell expansion in the root development. This study reports on the identification of AAK6 as a factor of quantitative root growth control, using phenotypic and genetic variation of natural populations of *Arabidopsis thaliana*. Our results demonstrate that AAK6 is required for normal cell production in the root meristematic zone and is consistent with repression of excessive cell expansion in the elongation zone. We have thereby characterized a case of a CCE in an organ of indeterminate growth (the root). Furthermore, the orthologue of *AAK6* encodes the well‐characterized yeast ribosome assembly factor Fap7 (Loc'h *et al.*, 2014; Peña *et al.*, 2016). Our data suggest that the molecular function of AK6 has been conserved between yeast and plants, Arabidopsis AK6 is involved in the assembly of ribosomes, and its natural alleles confer differential root growth.

### Controlling seed size leads to the identification of higher number of genome‐wide significant associations

High variance within samples reduces the statistical power of hypothesis testing and obscures the detection of growth differences between samples when they truly are present. As such, the variance of seed sizes impacts early root growth traits (this study; Elwell *et al.* 2011) and complicated identification of quantitative root growth factors. To reduce the variance of root growth as a result of variance in seed sizes, we used seeds of relatively homogeneous class (250–280 μm). Controlling the variance of seed sizes led to the identification of 138 genome‐wide significant associations across all the 78 studied traits cumulatively (14 morphology traits and two growth rate traits, each analysed during a 5‐d‐long growth assay; see Table S5, *Spreadsheet 1*). When we downsize the dataset from 252 to 160 accessions, present in our previous early‐root‐growth study (comparability described in the Materials and Methods section), then controlling the variance of seed sizes leads to the identification of 76 genome‐wide significant associations (Table S5, *Spreadsheet 2*) in contrast to 35 associations (Slovak *et al.*, 2014); these counts refer to the genome‐wide significant associations across all the 78 studied traits cumulatively. Furthermore, this is also indicated by the GWAS association that led to the identification of *AAK6* – while the most significantly associated *AAK6* marker SNP displayed a *P*‐value of 0.1889936 in a previous dataset that did not control seed size (Slovak *et al.*, 2014), the current seed size‐controlled dataset led to an association of this *AAK6* SNP with a *P*‐value of 0.0000223 (downsized seed size‐controlled dataset, *P* = 0.0019019). In conclusion, controlling seed size can be beneficial to decrease the variance in early root growth traits that was present as a result of maternal and seed size effects. The limitation of controlling seed size before a growth assay is that we will probably miss out on the identification of genetic loci controlling seed size and the studied root growth trait simultaneously. Overall, we find that this practice can decrease the variance of early root growth traits within (and between) accessions, can increase estimated heritability of the studied traits (Table S4), and can lead to the identification of considerably higher numbers of genome‐wide significant associations.

### Perturbation of ribosome biogenesis can lead to compensatory cell enlargement

We observed decreased cell production in the *aak6* root meristematic zone that is partially compensated for by an increased postmitotic cellular growth in the elongation zone and results in significantly longer mature root cells. Such compensation of the decrease in cell number by excessive cell enlargement was previously characterized in plant organs of determinate growth – the leaves (Tsukaya, 2003; Fujikura *et al.*, 2009). Here, we have shown unexpected evidence of CCE in the Arabidopsis root, an organ that continues to grow throughout the plant's lifetime. Furthermore, our findings are consistent with observed postmitotic cell enlargement being a consequence of a loss‐of‐function mutation of a gene which we show impacts ribosome abundance. While a previous study linked CCE in plants with ribosome‐related processes (Fujikura *et al.*, 2009), it relied on the annotation of yeast orthologues of Arabidopsis *oligocellula *(*oli*) genes. In this study we provide experimental evidence of altered plant ribosome abundance and CCE in the *aak6* mutant. Our evidence for the induction of CCE in a mutant of the ribosomal assembly factor gene *AAK6* is in agreement with previous evidence of CCE in *oli2 oli5* and *oli2 oli7* double mutants (Fujikura *et al.*, 2009) (genes orthologous to yeast ribosomal assembly and structural proteins encoding genes *Nop2*,* RPL5A* and *RPL5B*). This strongly suggests that perturbation of ribosome biogenesis can lead to CCE and that efficiency of ribosome biogenesis plays a role in cell production and determination of sizes of mature cells and plant organs. Given the large scale of the eukaryotic ribosome assembly machinery (reviewed by Peña *et al.*, 2017; Weis *et al.*, 2015), we can therefore hypothesize that additional genes involved in ribosome biogenesis will be identified, that mutations in these genes are likely to reduce cell production and that this in turn will lead to CCE in the respective mutant roots.

### The role of AAK6 in ribosome maturation and its link to root growth

While a previous study reported an *in vitro* protein interaction of Arabidopsis AK6 with the AtRPS14 ribosomal protein (Feng *et al.*, 2012), the role of AK6 in plants remained largely ill‐defined. Well‐studied homologous proteins of AAK6 (Fap7/aFap7) provide potential clues for a discussion of its possible mechanisms of action. AK6 interacts with the small ribosomal subunit (SSU) protein Rps14 in humans, yeast and archaea (Granemann *et al.*, 2005; Zhang *et al.*, 2012; Hellmich *et al.*, 2013). In yeast and archaea, its binding to uS11/RPS14 occludes the uS11/RPS14 RNA‐binding interface, suggesting that Fap7/aFap7 stabilizes uS11/RPS14 before being incorporated into pre‐SSU during ribosome biogenesis (Hellmich *et al.*, 2013; Loc'h *et al.*, 2014). This interaction is proposed to facilitate conformational change of the maturing SSU (Loc'h *et al.*, 2014). Recent genetic, biochemical and structural data indicate that Fap7 contributes to induction of a rotated state during pre‐SSU subunit maturation, thereby facilitating the release of the essential assembly factor Dim1 from pre‐SSU subunits late in their maturation (Ghalei *et al.*, 2017; Rai *et al.*, 2019). Lastly, Fap7, together with other assembly factors and ribosomal structural proteins, mediates the Nob1 executed SSU rRNA maturation (Granneman *et al.*, 2005; Strunk *et al.*, 2012), indicating that these proteins play a role in correct positioning of rRNA cleavage site with respect to the Nob1 nuclease active site. The final steps of ribosome biogenesis in yeast involve the joining of a pre‐SSU with a large ribosomal subunit to form an 80S complex in which final SSU maturation steps take place (Lebaron *et al.*, 2012; Strunk *et al.*, 2012). Ribosomal subunits are then dissociated before they enter the translational pool. Several defects in SSU subunit maturation as a result of assembly factor depletion (e.g. Fap7 and Rio1) and incomplete assembly cause overaccumulation of 80S ribosomes (Strunk *et al.*, 2012; Ferreira‐Cerca *et al.*, 2014; Loc'h *et al.*, 2014). Importantly, in agreement with these previous findings, we have shown that plants lacking AAK6 overaccumulate 80S ribosomes relative to polysome levels (Figs 7, S7) and this is consistent with AAK6 role in ribosome maturation. It therefore seems reasonable to assume that mechanisms of AK6 action described in yeast might be relevant for Arabidopsis. Nevertheless, further experiments will be required to test whether any of the roles of yeast AAK6 homologue (Fap7) (i.e. facilitation of a conformational change during ribosome assembly/maturation) are conserved in plants.

We propose that the observed root growth defects of *aak6* plants are a result of costs of overproduction of 80S ribosomes and that this 80S overaccumulation depletes organismal resources (nucleotides, rRNA, amino acids, ribosomal proteins as well as energy and molecular machinery used for their production) that cannot be utilized and turned into cell production. Consistently, an inverse relationship between ribosome abundance and biomass of Arabidopsis accessions was shown previously (Ishihara *et al.*, 2017). A higher protein turnover and the connected costs were suggested as the likely cause.

It remains to be established whether the mRNA‐free 80S ribosome quality control checkpoint that precedes translation, characterized in yeasts, exists in plants as well, but the *aak6*‐driven overaccumulation of 80S ribosomes suggests this possibility. Moreover, our results suggest that AK6 function in the final steps of ribosome assembly is conserved between yeast and Arabidopsis.

### Natural variation of AAK6 coding sequence probably impacts the efficiency of ribosome assembly

How, then, does the AAK6 function relate to effects of its natural allelic variants? The data presented here show that natural *AAK6* alleles modulate root growth of complemented *aak6* plants. While we did not directly investigate the molecular mechanism responsible for these allelic effects, we have gained some hints as to likely mechanisms. There are several polymorphisms in the noncoding sequences in the genomic fragments of *AAK6*; however, we did not find any correlation with steady‐state levels of *AAK6* mRNA and growth rate among several tested accessions. Moreover, the ectopic overexpression of *AAK6* does not significantly impact root growth in Arabidopsis (Fig. S2b). This is consistent with the wild‐type‐like phenotype of the Fap7 overexpression line in yeast (Juhnke *et al.*, 2000). We therefore propose that the need for AAK6 abundance is saturated in Arabidopsis accessions and that it is rather the natural genetic variation in *AAK6* coding region that affects the AAK6 function and leads to root growth differences. Although the four nonsynonymous amino acid substitutions in the coding region of *AAK6* genomic fragments used for *aak6* complementation are the prime candidates for the functional basis for observed variation, we cannot exclude differential post‐transcriptional regulation of natural *AAK6* alleles either. We hypothesize that the natural variation modulates the efficiency of AAK6‐facilitated ribosome assembly, which impacts cell production and finally root growth.

## Author contributions

RS and WB conceived the study; RS, MR and JB developed the methodology and carried out the formal analysis; RS, CS, MR and JB carried out the investigation; CS and KJ were responsible for resources and data curation; CG, RS, JB, GTSB and WB developed and operated the software; GTSB and WB supervised the research; and RS and WB wrote the manuscript.

## Supporting information

Please note: Wiley Blackwell are not responsible for the content or functionality of any Supporting Information supplied by the authors. Any queries (other than missing material) should be directed to the *New Phytologist* Central Office.
**Fig. S1** Natural phenotypic variation and GWAS of root growth rate 7^th^ day after stratification.
**Fig. S2** Relative *AAK6* expression levels and root growth in wild‐type, SALK_015289 (*aak6* ‐/‐) and *AAK6* overexpression line (*35S:AAK6*).
**Fig. S3** Complementation of *aak6* *‐/‐* by *pAAK6:AAK6‐Clover*, in the shoots.
**Fig. S4** Complementation of *aak6 ‐/‐* by *pAAK6:AAK6*, in the root.
**Fig. S5** Root anatomy parameters of *aak6 ‐/‐ *compared with the wild‐type.
**Fig. S6** Kinematic analysis of root growth.
**Fig. S7** Replicates of ribosome/polysome profiles.
**Methods S1** Relative expression fold change of the *AAK6* gene.
**Methods S2** Seed size measurements.
**Notes S1** Precautions taken in propagation of natural accessions.
**Notes S2** Precautions taken with SALK T‐DNA lines.
**Notes S3** Increase in endopolyploidy levels accompanies an increase in mature cell size in the *aak6* mutant.
**Table S1** 252 *Arabidopsis thaliana* accessions used in this study.
**Table S2** Phenotypes of T‐DNA insertion mutant lines (*t*‐test, *P*‐values).
**Table S3** Primers used in this study.
**Table S4** Impact of seed size on root growth traits.
**Table S5** Genome‐wide significant GWAS associations (FDR 5%) aggregated for 16 root growth traits and time‐course experiment, 3^rd^ – 7^th^ day after stratification (DAS).
**Table S6** Alignment of *AAK6* coding sequences (amino acids) of Bå4‐1, Col‐0 and Tha‐1.
**Table S7** Proportions of nuclear ploidy classes in the root apices.

## References

[nph16291-bib-0001] Bai D , Zhang J , Li T , Hang R , Liu Y , Tian Y , Huang D , Qu L , Cao X , Ji J , Zheng X . 2016 The ATPase hCINAP regulates 18S rRNA processing and is essential for embryogenesis and tumour growth. Nature Communications 7: 12310. 10.1038/ncomms12310 PMC4974663 27477389

[nph16291-bib-0002] Beemster GTS , Baskin TI . 1998 Analysis of cell division and elongation underlying the developmental acceleration of root growth in *Arabidopsis thaliana* . Plant Physiology 116: 1515–1526. 9536070 10.1104/pp.116.4.1515 PMC35060

[nph16291-bib-0003] Barakat A , Szick‐Miranda K , Chang IF , Guyot R , Blanc G , Cooke R , Delseny M , Bailey‐Serres J . 2001 The organization of cytoplasmic ribosomal protein genes in the Arabidopsis genome. Plant Physiology 127: 398–415. 11598216 PMC125077

[nph16291-bib-0004] Brooks RF . 1977 Continuous protein synthesis is required to maintain the probability of entry into S phase. Cell 12: 311–317. 902318 10.1016/0092-8674(77)90209-4

[nph16291-bib-0005] Byrne ME . 2009 A role for the ribosome in development. Trends in Plant Science 14: 512–519. 19716746 10.1016/j.tplants.2009.06.009

[nph16291-bib-0006] Chaiwanon J , Wang ZY . 2015 Spatiotemporal brassinosteroid signaling and antagonism with auxin pattern stem cell dynamics in Arabidopsis roots. Current Biology 25: 1031–1042. 25866388 10.1016/j.cub.2015.02.046 PMC4415608

[nph16291-bib-0007] De Schutter K , Joubès J , Cools T , Verkest A , Corellou F , Babiychuk E , Van Der Schueren E , Beeckman T , Kushnir S , Inzé D , De Veylder L . 2007 Arabidopsis WEE1 kinase controls cell cycle arrest in response to activation of the DNA integrity checkpoint. Plant Cell 19: 211–225. 17209125 10.1105/tpc.106.045047 PMC1820959

[nph16291-bib-0008] De Veylder L , Beeckman T , Beemster GTS , Krols L , Terras F , Landrieu I , Van Der Schueren E , Maes S , Naudts M , Inzé D . 2001 Functional analysis of cyclin‐dependent kinase inhibitors of Arabidopsis. Plant Cell 13: 1653–1668. 11449057 10.1105/TPC.010087 PMC139548

[nph16291-bib-0009] Elwell AL , Gronwall DS , Miller ND , Spalding EP , Brooks TLD . 2011 Separating parental environment from seed size effects on next generation growth and development in Arabidopsis. Plant, Cell & Environment 34: 291–301. 10.1111/j.1365-3040.2010.02243.x 20955226

[nph16291-bib-0010] Emami S , Mc Yee , Dinneny JR . 2013 A robust family of Golden Gate *Agrobacterium* vectors for plant synthetic biology. Frontiers in Plant Science 4: 339. 24032037 10.3389/fpls.2013.00339 PMC3759027

[nph16291-bib-0011] Fantes P , Nurse P . 1977 Control of cell size at division in fission yeast by a growth‐modulated size control over nuclear division. Experimental Cell Research 107: 377–386. 872891 10.1016/0014-4827(77)90359-7

[nph16291-bib-0012] Feng X , Yang R , Zheng X , Zhang F . 2012 Identification of a novel nuclear‐localized Adenylate Kinase 6 from *Arabidopsis thaliana* as an essential stem growth factor. Plant Physiology and Biochemistry 61: 180–186. 23121860 10.1016/j.plaphy.2012.10.002

[nph16291-bib-0013] Ferjani A , Horiguchi G , Yano S , Tsukaya H . 2007 Analysis of leaf development in *fugu* mutants of Arabidopsis reveals three compensation modes that modulate cell expansion in determinate organs. Plant Physiology 144: 988–999. 17468216 10.1104/pp.107.099325 PMC1914195

[nph16291-bib-0014] Ferreira‐Cerca S , Kiburu I , Thomson E , LaRonde N , Hurt E . 2014 Dominant Rio1 kinase/ATPase catalytic mutant induces trapping of late pre‐40S biogenesis factors in 80S‐like ribosomes. Nucleic Acids Research 42: 8635–8647. 24948609 10.1093/nar/gku542 PMC4117770

[nph16291-bib-0015] Fujikura U , Horiguchi G , Ponce MR , Micol JL , Tsukaya H . 2009 Coordination of cell proliferation and cell expansion mediated by ribosome‐related processes in the leaves of *Arabidopsis thaliana* . Plant Journal 59: 499–508. 19392710 10.1111/j.1365-313X.2009.03886.x

[nph16291-bib-0016] Ghalei H , Trepreau J , Collins JC , Bhaskaran H , Strunk BS , Karbstein K . 2017 The ATPase Fap7 tests the ability to carry out translocation‐like conformational changes and releases Dim1 during 40S ribosome maturation. Molecular Cell 6: 990–1000. 10.1016/j.molcel.2017.08.007 PMC6192259 28890337

[nph16291-bib-0017] Granneman S , Nandineni MR , Baserga SJ . 2005 The putative NTPase Fap7 mediates cytoplasmic 20S pre‐rRNA processing through a direct interaction with Rps14. Molecular and Cellular Biology 25: 10352–10364. 16287850 10.1128/MCB.25.23.10352-10364.2005 PMC1291222

[nph16291-bib-0018] Griffith ME , Mayer U , Capron A , Ngo QA , Surendrarao A , McClinton R , Jürgens G , Sundaresan V . 2007 The *TORMOZ* gene encodes a nucleolar protein required for regulated division planes and embryo development in Arabidopsis. Plant Cell 19: 2246–2263. 17616738 10.1105/tpc.106.042697 PMC1955705

[nph16291-bib-0019] Guan Q , Wu J , Yue X , Zhang Y , Zhu J . 2013 A nuclear calcium‐sensing pathway is critical for gene regulation and salt stress tolerance in Arabidopsis. PLoS Genetics 9: e1003755. 24009530 10.1371/journal.pgen.1003755 PMC3757082

[nph16291-bib-0020] Hayashi K , Hasegawa J , Matsunaga S . 2013 The boundary of the meristematic and elongation zones in roots: endoreduplication precedes rapid cell expansion. Scientific Reports 3: 2723. 24121463 10.1038/srep02723 PMC3796303

[nph16291-bib-0021] Hellens RP , Edwards EA , Leyland NR , Bean S , Mullineaux PM . 2000 pGreen: a versatile and flexible binary Ti vector for *Agrobacterium*‐mediated plant transformation. Plant Molecular Biology 42: 819–832. 10890530 10.1023/a:1006496308160

[nph16291-bib-0071] Hellmich UA , Weis BL , Lioutikov A , Wurm JP , Kaiser M , Christ NA , Hantke K , Kötter P , Entian KD , Schleiff E *et al.* 2013 Essential ribosome assembly factor Fap7 regulates a hierarchy of RNA–protein interactions during small ribosomal subunit biogenesis. Proceedings of the National Academy of Sciences, USA 110: 15253–15258. 10.1073/pnas.1306389110 PMC3780860 24003121

[nph16291-bib-0022] Hemerly A , Engler JdA , Bergounioux C , Van Montagu M , Engler G , Inzé D , Ferreira P . 1995 Dominant negative mutants of the Cdc2 kinase uncouple cell division from iterative plant development. EMBO Journal 14: 3925–3936. 7664733 10.1002/j.1460-2075.1995.tb00064.x PMC394471

[nph16291-bib-0023] Horváth BM , Magyar Z , Zhang Y , Hamburger AW , Bakó L , Visser RGF , Bachem CWB , Bögre L . 2006 EBP1 regulates organ size through cell growth and proliferation in plants. EMBO Journal 25: 4909–4920. 17024182 10.1038/sj.emboj.7601362 PMC1618091

[nph16291-bib-0024] Inagaki S , Umeda M . 2011 Cell‐cycle control and plant development. International Review of Cell and Molecular Biology 291: 227–261. 22017978 10.1016/B978-0-12-386035-4.00007-0

[nph16291-bib-0025] Ishida T , Adachi S , Yoshimura M , Shimizu K , Umeda M , Sugimoto K . 2009 Auxin modulates the transition from the mitotic cycle to the endocycle in Arabidopsis. Development 137: 63–71. 10.1242/dev.035840 20023161

[nph16291-bib-0026] Ishihara H , Moraes TA , Pyl ET , Schulze WX , Obata T , Scheffel A , Fernie AR , Sulpice R , Stitt M . 2017 Growth rate correlates negatively with protein turnover in Arabidopsis accessions. The Plant Journal 91: 416–429. 28419597 10.1111/tpj.13576

[nph16291-bib-0027] Johnston GC , Pringle JR , Hartwell LH . 1977 Coordination of growth with cell division in the yeast *Saccharomyces cerevisiae* . Experimental Cell Research 105: 79–98. 320023 10.1016/0014-4827(77)90154-9

[nph16291-bib-0028] Juhnke H , Charizanis C , Latifi F , Krems B , Entian KD . 2000 The essential protein fap7 is involved in the oxidative stress response of *Saccharomyces cerevisiae* . Molecular Microbiology 35: 936–948. 10692169 10.1046/j.1365-2958.2000.01768.x

[nph16291-bib-0029] Kafri M , Metzl‐Raz E , Jona G , Barkai N . 2016 The cost of protein production. Cell Reports 14: 22–31. 26725116 10.1016/j.celrep.2015.12.015 PMC4709330

[nph16291-bib-0030] Kang HM , Sul JH , Service SK , Zaitlen NA , Kong SY , Freimer NB , Sabatti C , Eskin E . 2010 Variance component model to account for sample structure in genome‐wide association studies. Nature Genetics 42: 348–354. 20208533 10.1038/ng.548 PMC3092069

[nph16291-bib-0031] Kang HM , Na Zaitlen , Wade CM , Kirby A , Heckerman D , Daly MJ , Eskin E . 2008 Efficient control of population structure in model organism association mapping. Genetics 178: 1709–1723. 18385116 10.1534/genetics.107.080101 PMC2278096

[nph16291-bib-0032] Klepikova AV , Kasianov AS , Gerasimov ES , Logacheva MD , Penin AA . 2016 A high resolution map of the *Arabidopsis thaliana* developmental transcriptome based on RNA‐seq profiling. The Plant Journal 88: 1058–1070. 27549386 10.1111/tpj.13312

[nph16291-bib-0033] Kojima H , Suzuki T , Kato T , Ki E , Sato S , Kato T , Tabata S , Saez‐Vasquez J , Echeverria M , Nakagawa T , Ishiguro S , Nakamura K . 2007 Sugar‐inducible expression of the *nucleolin‐1* gene of *Arabidopsis thaliana* and its role in ribosome synthesis, growth and development. The Plant Journal 49: 1053–1063. 17286797 10.1111/j.1365-313X.2006.03016.x

[nph16291-bib-0034] Lebaron S , Schneider C , van Nues RW , Swiatkowska A , Walsh D , Bottcher B , Granneman S , Watkins NJ , Tollervey D . 2012 Proofreading of pre‐40S ribosome maturation by a translation initiation factor and 60S subunits. Nature Structural and Molecular Biology 19: 744–753. 10.1038/nsmb.2308 PMC3654374 22751017

[nph16291-bib-0035] Liebermeister W , Noor E , Flamholz A , Davidi D , Bernhardt J , Milo R . 2014 Visual account of protein investment in cellular functions. Proceedings of the National Academy of Sciences, USA 111: 8488–8493. 10.1073/pnas.1314810111 PMC4060655 24889604

[nph16291-bib-0036] Loc'h J , Blaud M , Réty S , Lebaron S , Deschamps P , Bareille J , Jombart J , Robert‐Paganin J , Delbos L , Chardon F , Zhang E , Charenton C , Tollervey D , Leulliot N . 2014 RNA mimicry by the Fap7 adenylate kinase in ribosome biogenesis. PLoS Biology 12: e1001860. 24823650 10.1371/journal.pbio.1001860 PMC4019466

[nph16291-bib-0037] Martin SG , Berthelot‐Grosjean M . 2009 Polar gradients of the DYRK‐family kinase Pom1 couple cell length with the cell cycle. Nature 459: 852–856. 19474792 10.1038/nature08054

[nph16291-bib-0038] Mitchison JM , Creanor J . 1971 Induction synchrony in the fission yeast *Schizosaccharomyces pombe* . Experimental Cell Research 67: 368–374. 4255493 10.1016/0014-4827(71)90421-6

[nph16291-bib-0039] Moseley JB , Mayeux A , Paoletti A , Nurse P . 2009 A spatial gradient coordinates cell size and mitotic entry in fission yeast. Nature 459: 857–860. 19474789 10.1038/nature08074

[nph16291-bib-0040] Moubayidin L , Perilli S , Dello Ioio R , Di Mambro R , Costantino P , Sabatini S . 2010 The rate of cell differentiation controls the Arabidopsis root meristem growth phase. Current Biology 20: 1138–1143. 20605455 10.1016/j.cub.2010.05.035

[nph16291-bib-0041] Neufeld TP , de la Cruz AF , Johnston LA , Edgar BA . 1998 Coordination of growth and cell division in the *Drosophila* wing. Cell 93: 1183–1193. 9657151 10.1016/s0092-8674(00)81462-2

[nph16291-bib-0042] Peña C , Hurt E , Panse VG . 2017 Eukaryotic ribosome assembly, transport and quality control. Nature Structural & Molecular Biology 24: 689–699. 10.1038/nsmb.3454 28880863

[nph16291-bib-0043] Peña C , Schütz S , Fischer U , Chang Y , Panse VG . 2016 Prefabrication of a ribosomal protein subcomplex essential for eukaryotic ribosome formation. eLife 5: e21755. 27929371 10.7554/eLife.21755 PMC5148605

[nph16291-bib-0044] Petricka JJ , Nelson TM . 2007 Arabidopsis nucleolin affects plant development and patterning. Plant Physiology 144: 173–186. 17369435 10.1104/pp.106.093575 PMC1913809

[nph16291-bib-0045] Polymenis M , Aramayo R . 2015 Translate to divide: control of the cell cycle by protein synthesis. Microbial Cell 2: 94–104. 28357283 10.15698/mic2015.04.198 PMC5348972

[nph16291-bib-0046] Preibisch S , Saalfeld S , Tomancak P . 2009 Globally optimal stitching of tiled 3D microscopic image acquisitions. Bioinformatics 25: 1463–1465. 19346324 10.1093/bioinformatics/btp184 PMC2682522

[nph16291-bib-0047] Rai J , Parker MD , Ghalei H , Johnson MC , Karbstein K , Stroupe ME . 2019 Subunit joining exposes nascent pre‐40S rRNA for processing and quality control. bioRxiv. 10.1101/617910.

[nph16291-bib-0048] Randall RS , Sornay E , Dewitte W , Murray JAH . 2015 *AINTEGUMENTA* and the D‐type cyclin CYCD3;1 independently contribute to petal size control in Arabidopsis: evidence for organ size compensation being an emergent rather than a determined property. Journal of Experimental Botany 66: 3991–4000. 25948704 10.1093/jxb/erv200 PMC4473993

[nph16291-bib-0049] Roeder AHK , Chickarmane V , Cunha A , Obara B , Meyerowitz EM . 2010 Variability in the control of cell division underlies sepal epidermal patterning in *Arabidopsis thaliana* . PLoS Biology 8: e1000367. 20485493 10.1371/journal.pbio.1000367 PMC2867943

[nph16291-bib-0050] Rolfe DF , Brown GC . 1997 Cellular energy utilization and molecular origin of standard metabolic rate in mammals. Physiology Reviews 77: 731–758. 10.1152/physrev.1997.77.3.731 9234964

[nph16291-bib-0051] Russell JB , Cook GM . 1995 Energetics of bacterial growth: balance of anabolic and catabolic reactions. Microbiology Reviews 59: 48–62. 10.1128/mr.59.1.48-62.1995 PMC239354 7708012

[nph16291-bib-0052] Satbhai SB , Setzer C , Freynschlag F , Slovak R , Kerdaffrec E , Busch W . 2017 Natural allelic variation of *FRO2* modulates Arabidopsis root growth under iron deficiency. Nature Communication 8: 15603. 10.1038/ncomms15603 PMC5458102 28537266

[nph16291-bib-0053] Schneider CA , Rasband WS , Eliceiri KW . 2012 NIH Image to ImageJ: 25 years of image analysis. Nature Methods 9: 671–675. 22930834 10.1038/nmeth.2089 PMC5554542

[nph16291-bib-0054] Seren Ü , Vilhjálmsson BJ , Horton MW , Meng D , Forai P , Huang YS , Long Q , Segura V , Nordborg M . 2012 GWAPP: a web application for genome‐wide association mapping in Arabidopsis. Plant Cell 24: 4793–805. 23277364 10.1105/tpc.112.108068 PMC3556958

[nph16291-bib-0055] Shi DQ , Liu J , Xiang YH , Ye D , Sundaresan V , Yang WC . 2005 SLOW WALKER1, essential for gametogenesis in Arabidopsis, encodes a WD40 protein involved in 18S ribosomal RNA biogenesis. Plant Cell 17: 2340–2354. 15980260 10.1105/tpc.105.033563 PMC1182493

[nph16291-bib-0056] Slovak R , Göschl Christian SuX , Shimotani K , Shiina T , Busch W . 2014 A scalable open‐source pipeline for large‐scale root phenotyping of Arabidopsis. Plant Cell 26: 2390–2403. 24920330 10.1105/tpc.114.124032 PMC4114940

[nph16291-bib-0057] Strunk BS , Novak MN , Young CL , Karbstein K . 2012 A translation‐like cycle is a quality control checkpoint for maturing 40S ribosome subunits. Cell 150: 111–121. 22770215 10.1016/j.cell.2012.04.044 PMC3615461

[nph16291-bib-0058] Trucco E . 1970 On the average cellular volume in synchronized cell populations. Bulletin of Mathematical Biophysics 32: 459–473. 5513388 10.1007/BF02476765

[nph16291-bib-0059] Tsukaya H . 2003 Organ shape and size: a lesson from studies of leaf morphogenesis. Current Opinion in Plant Biology 6: 57–62. 12495752 10.1016/s1369526602000055

[nph16291-bib-0060] Ubeda‐Tomás S , Beemster GTS , Bennett MJ . 2012 Hormonal regulation of root growth: integrating local activities into global behaviour. Trends in Plant Science 17: 326–331. 22401844 10.1016/j.tplants.2012.02.002

[nph16291-bib-0061] Ubeda‐Tomás S , Federici F , Casimiro I , Beemster GTS , Bhalerao R , Swarup R , Doerner P , Haseloff J , Bennett MJ . 2009 Gibberellin signaling in the endodermis controls arabidopsis root meristem size. Current Biology 19: 1194–1199. 19576770 10.1016/j.cub.2009.06.023

[nph16291-bib-0062] Unger MW , Hartwell LH . 1976 Control of cell division in *Saccharomyces cerevisiae* by methionyl‐tRNA. Proceedings of the National Academy of Sciences, USA 73: 1664–1668. 10.1073/pnas.73.5.1664 PMC430360 775494

[nph16291-bib-0063] Wang WH , Chen J , Liu TW , Chen J , Han AD , Simon M , Dong XJ , He JX , Zheng HL . 2014 Regulation of the calcium‐sensing receptor in both stomatal movement and photosynthetic electron transport is crucial for water use efficiency and drought tolerance in Arabidopsis. Journal of Experimental Botany 65: 223. 24187420 10.1093/jxb/ert362 PMC3883291

[nph16291-bib-0064] Warner JR . 1999 The economics of ribosome biosynthesis in yeast. Trends in Biochemical Science 24: 437–440. 10.1016/s0968-0004(99)01460-7 10542411

[nph16291-bib-0065] Weigel D , Glazebrook J . 2006 In planta transformation of Arabidopsis. CSH Protocols 2006: pdb.prot4668. 10.1101/pdb.prot4668 22484684

[nph16291-bib-0066] Weis BL , Kovacevic J , Missbach S , Schleiff E . 2015 Plant‐specific features of ribosome biogenesis. Trends in Plant Science 20: 729–740. 26459664 10.1016/j.tplants.2015.07.003

[nph16291-bib-0067] Woolford JL , Baserga SJ . 2013 Ribosome biogenesis in the yeast *Saccharomyces cerevisiae* . Genetics 195: 643–681. 24190922 10.1534/genetics.113.153197 PMC3813855

[nph16291-bib-0068] Worley MI , Setiawan L , Hariharan IK . 2013 TIE‐ DYE: a combinatorial marking system to visualize and genetically manipulate clones during development in Drosophila melanogaster. Development 140: 3275–3284. 23785055 10.1242/dev.096057 PMC3931737

[nph16291-bib-0069] Yang X , Dong G , Palaniappan K , Mi G , Baskin TI . 2017 Temperature‐compensated cell production rate and elongation zone length in the root of *Arabidopsis thaliana* . Plant, Cell & Environment 40: 264–276. 10.1111/pce.12855 27813107

[nph16291-bib-0070] Zhang J , Bai D , Ma X , Guan J , Zheng X . 2012 hCINAP is a novel regulator of ribosomal protein-HDM2-p53 pathway by controlling NEDDylation of ribosomal protein S14. Oncogene 33: 246–254. 23246961 10.1038/onc.2012.560

