# Supplementary material for: Ribosome assembly factor Adenylate Kinase 6 maintains cell proliferation and cell size homeostasis during root growth
Source: New Phytol. 2019 Dec 2;225(5):2064–76. doi: 10.1111/nph.16291 (PMC7028144; doi:10.1111/nph.16291)
Supplement: Supplementary file 1 — Please note: Wiley Blackwell are not responsible for the content or functionality of any Supporting Information supplied by the authors. Any queries (other than missing material) should be directed to the New Phytologist Central Office. Fig. S1 Natural phenotypic variation and GWAS of root growth rate 7th day after stratification. Fig. S2 Relative AAK6 expression levels and root growth in wild‐type, SALK_015289 (aak6 ‐/‐) and AAK6 overexpression line (35S:AAK6). Fig. S3 Complementation of aak6 ‐/‐ by pAAK6:AAK6‐Clover, in the shoots. Fig. S4 Complementation of aak6 ‐/‐ by pAAK6:AAK6, in the root. Fig. S5 Root anatomy parameters of aak6 ‐/‐ compared with the wild‐type. Fig. S6 Kinematic analysis of root growth. Fig. S7 Replicates of ribosome/polysome profiles. Methods S1 Relative expression fold change of the AAK6 gene. Methods S2 Seed size measurements. Notes S1 Precautions taken in propagation of natural accessions. Notes S2 Precautions taken with SALK T‐DNA lines. Notes S3 Increase in endopolyploidy levels accompanies an increase in mature cell size in the aak6 mutant. Table S1 252 Arabidopsis thaliana accessions used in this study. Table S2 Phenotypes of T‐DNA insertion mutant lines (t‐test, P‐values). Table S3 Primers used in this study. Table S4 Impact of seed size on root growth traits. Table S5 Genome‐wide significant GWAS associations (FDR 5%) aggregated for 16 root growth traits and time‐course experiment, 3rd – 7th day after stratification (DAS). Table S6 Alignment of AAK6 coding sequences (amino acids) of Bå4‐1, Col‐0 and Tha‐1. Table S7 Proportions of nuclear ploidy classes in the root apices. [file NPH-225-2064-s001.pdf]

Article title: Ribosome assembly factor Adenylate Kinase 6 maintains cell proliferation and cell size homeostasis during root growth

Authors: Radka Slovak, Claudia Setzer, Mykola Roiuk, Jonas Bertels, Christian Göschl, Katharina Jandrasits, Gerrit T. S. Beemster, and Wolfgang Busch

Article acceptance date: 19 October 2019.

The following Supplementary Data is available for this article:

Fig. S1 Natural phenotypic variation and GWAS of root growth rate 7th day after stratification

Fig. S2 Relative *AAK6* expression levels and root growth in wild type, SALK_015289 (*aak6* -/-) and *AAK6* overexpression line (*35S:AAK6*)

Fig. S3 Complementation of *aak6* -/- by *pAAK6:AAK6-Clover*, in the shoots

Fig. S4 Complementation of *aak6* -/- by *pAAK6:AAK6*, in the root

Fig. S5 Root anatomy parameters of *aak6* -/- compared to wild type

Fig. S6 Kinematic analysis of root growth

Fig. S7 Replicates of ribosome/polysome profiles

Table S1 252 *Arabidopsis thaliana* accessions used in this study

Table S2 Phenotypes of T-DNA insertion mutant lines (t-test p-values)

Table S3 Primers used in this study

Table S4 Impact of seed size on root growth traits

Table S5 Genome-wide significant GWAS associations (FDR 5%) aggregated for 16 root growth traits and time-course experiment, 3rd - 7th day after stratification (DAS). *Spreadsheet 1*: Data from 252 accessions. *Spreadsheet 2*: Data from 160 accessions

Table S6 Alignment of *AAK6* coding sequences (amino acids) of Bå4-1, Col-0, and Tha-1

Table S7 Proportions of nuclear ploidy classes in the root apices

Methods S1 Relative expression fold change of the *AAK6* gene

Methods S2 Seed size measurements

Notes S1 Precautions taken in propagation of natural accessions

Notes S2 Precautions taken with SALK T-DNA lines

Notes S3 Increase in endopolyploidy levels accompanies increase in mature cell size in *aak6* mutant

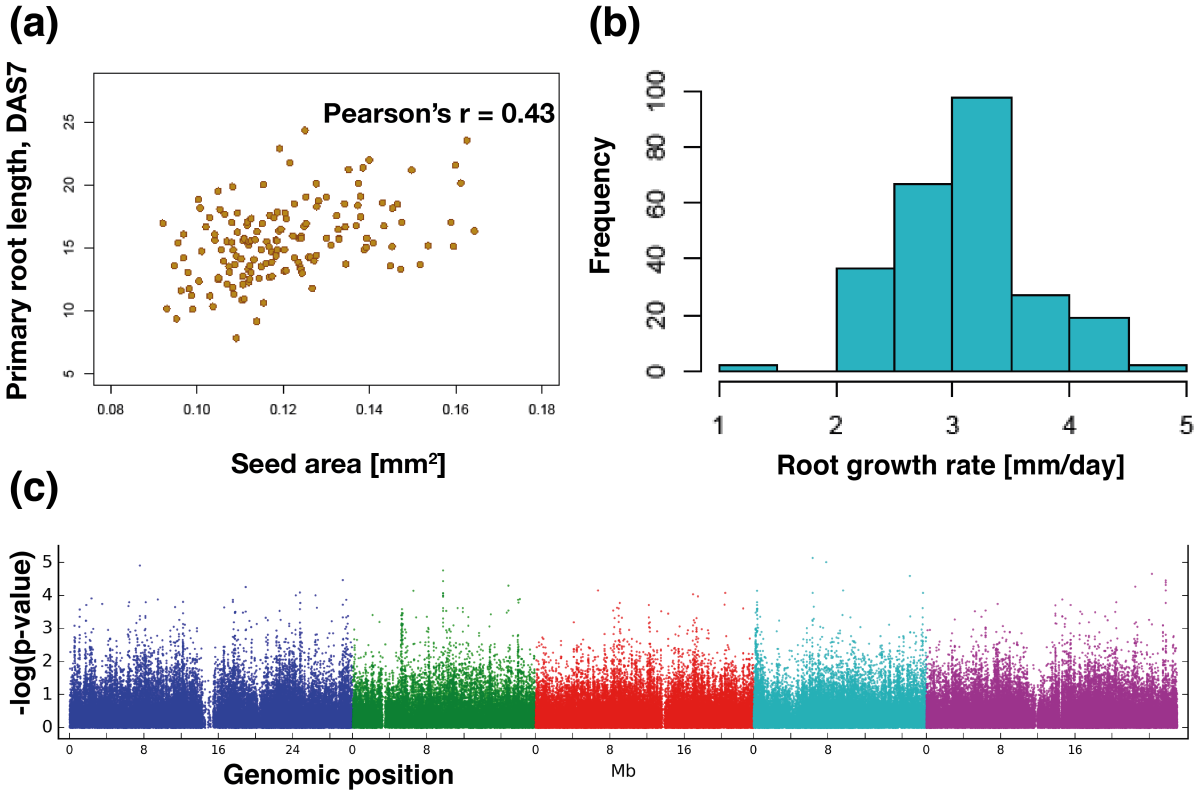

(d)

Chromosome	SNP pos	GWAS p-value	-log(p-value)	Gene start	Gene end	Gene orientation	Distance from TSS	Distance from gene	Relative position	AGI	Short name
1	7566805	1.23E-05	4.9	7557262	7565803	-	-1002	-1002	upstream	AT1G21580	
1	7566805	1.23E-05	4.9	7566232	7569902	-	3097	in gene	in gene	AT1G21590	
2	9754370	1.75E-05	4.8	9749869	9752863	+	4501	1507	downstream	AT2G22910	NAGS1
2	9754370	1.75E-05	4.8	9753631	9757600	+	739	in gene	in gene	AT2G22920	SCPL12
4	6341639	7.22E-06	5.1	6344331	6345537	+	-2692	-2692	upstream	AT4G10170	
4	7816687	9.81E-06	5.0	7812745	7813967	+	3942	2720	downstream	AT4G13442	
4	7816687	9.81E-06	5.0	7814202	7814274	+	2485	2413	downstream	AT4G13445	tRNA
4	7816687	9.81E-06	5.0	7814960	7816004	-	-683	1727	upstream	AT4G13450	
5	24278248	2.23E-05	4.7	24273879	24274551	-	-3697	-3697	upstream	AT5G60335	
5	24278248	2.23E-05	4.7	24274962	24276055	-	-2193	-2193	upstream	AT5G60340	AAK6
5	24278248	2.23E-05	4.7	24277730	24279147	+	518	in gene	in gene	AT5G60350	
5	24278248	2.23E-05	4.7	24279897	24282397	+	-1649	-1649	upstream	AT5G60360	SAG2

Fig. S1 Natural phenotypic variation and GWAS of root growth rate 7th day after stratification. (a) Correlation of average root length on 7th day after stratification (DAS) (seedlings from seeds of natural seed sizes (Slovak et al., 2014)) with average seed size (this study, Methods S2) for 160 natural accessions. Each dot represents one accession. (b) Histogram of natural phenotypic variation of root growth rate on DAS7 (seedlings from size-controlled seeds) (c) GWAS Manhattan plot showing association of SNPs and root growth rate on DAS7 data from 252 accessions; x-axis: genomic position, y-axis: -log(p-value) for each SNP. (*D*) TOP 5 associated genomic regions for GWAS of root growth rate on DAS7.

Slovak R, Göschl Christian, Su X, Shimotani K, Shiina T and Busch W. 2014. A Scalable Open-Source Pipeline for Large-Scale Root Phenotyping of Arabidopsis. Plant Cell 26: 2390–2403.

(a)

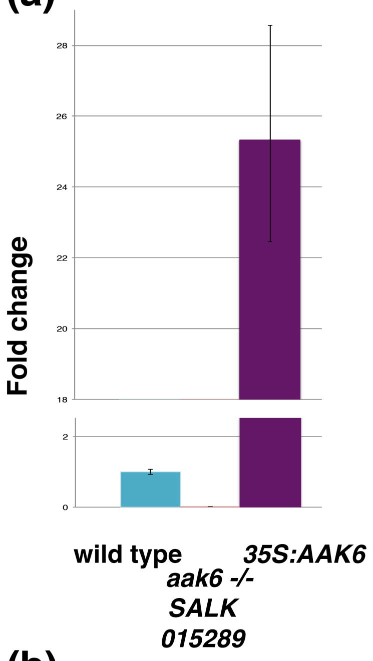

(b)

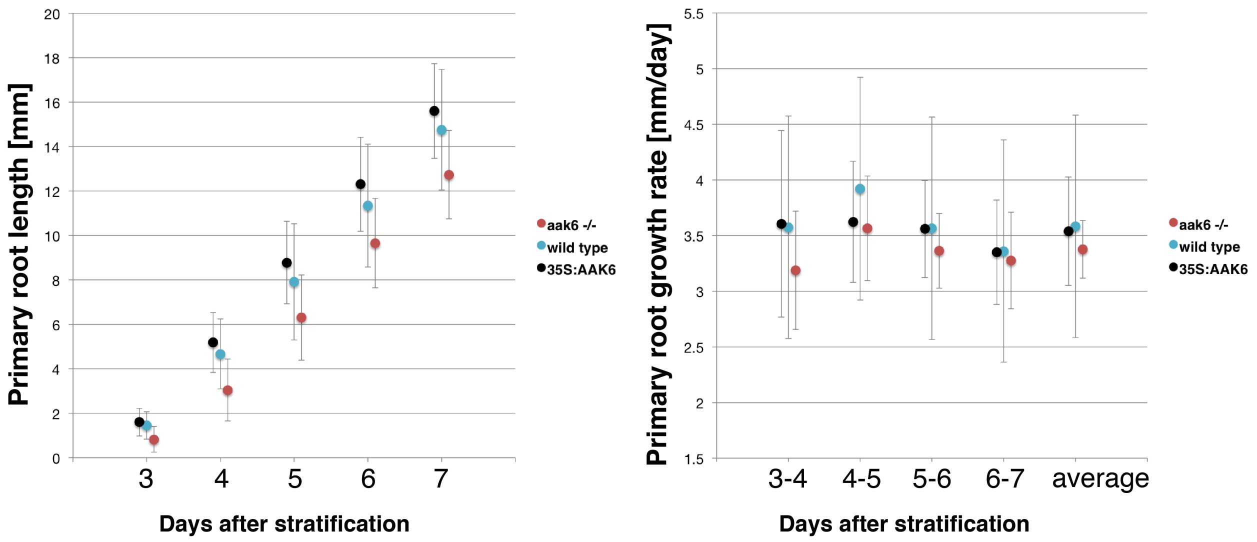

	TL DAS3	TL DAS4	TL DAS5	TL DAS6	TL DAS7	GR DAS 3-4	GR DAS 4-5	GR DAS 5-6	GR DAS 6-7	GR average
**Col-0 vs. aak6**	0.006	0.000	0.008	0.011	0.001	0.132	0.022	0.045	0.439	**0.027**
**Col-0 vs. OX**	0.386	0.180	0.146	0.132	0.173	0.896	0.069	0.967	0.929	0.695

in blue: values highlighted at an uncorrected significance treshold alpha = 0.05

in red: significant after correction for testing multiple genotypes, significance treshold alpha = 0.025

Fig. S2 Relative *AAK6* expression levels and root growth in the wild type, SALK_015289 (*aak6 -/-*) and *AAK6* overexpression line (*35S:AAK6*). (a) Relative *AAK6* expression levels in the wild type, SALK_015289 (*aak6 -/-*) and *AAK6* overexpression line (*35S:AAK6*), whiskers represent standard deviation. See also Methods S1. (b) Root growth of *aak6*, wild type and *35S:AAK6*, 3rd - 7th day after stratification (DAS). Whiskers represent standard deviation. t-test p-values are indicated in the table.

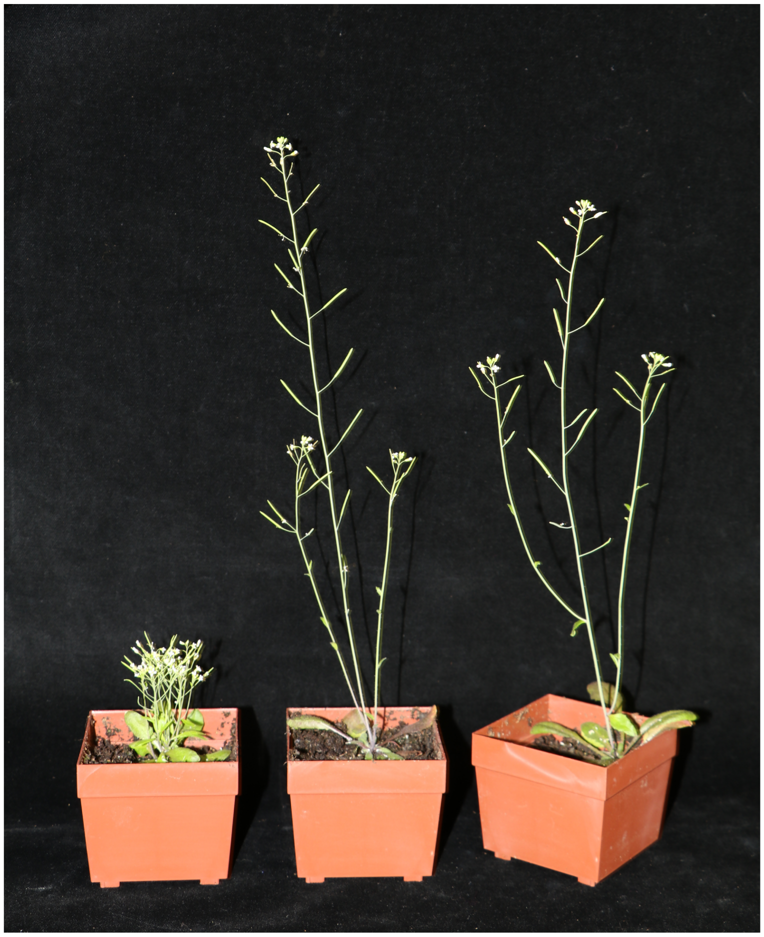

**transgene:**		***pAAK6:AAK6-Clover***	***pAAK6:AAK6-Clover***
**background:**	**aak6 -/-**	**aak6 -/-**	**wild type**

Fig. S3 Complementation of *aak6 -/-* by *pAAK6:AAK6-Clover*, in the shoots. 2 month old representative plants.

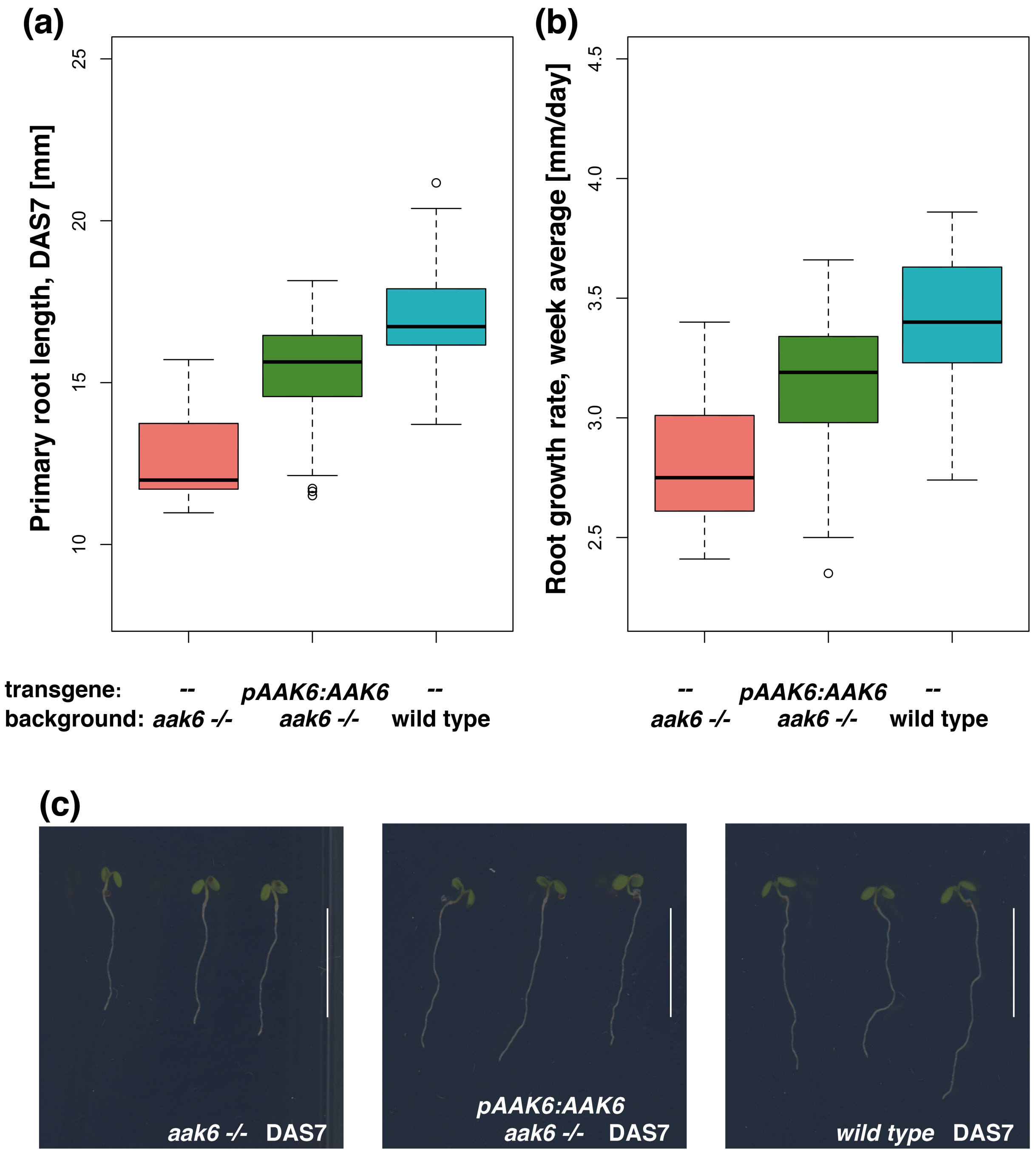

Fig. S4 Complementation of *aak6 -/-* by *pAAK6: AAK6*, in the root. (*a*) Primary root length of seedlings on 7th day after stratification. Whiskers represent the last value at a distance smaller than 1.5 times the interquartile range (IQR). (*b*) Root growth rate of seedlings averaged over 3rd - 7th day after stratification. Whiskers < 1.5 × IQR. (*c*) Representative examples of seedlings. Scale bar 1 cm.

(a)

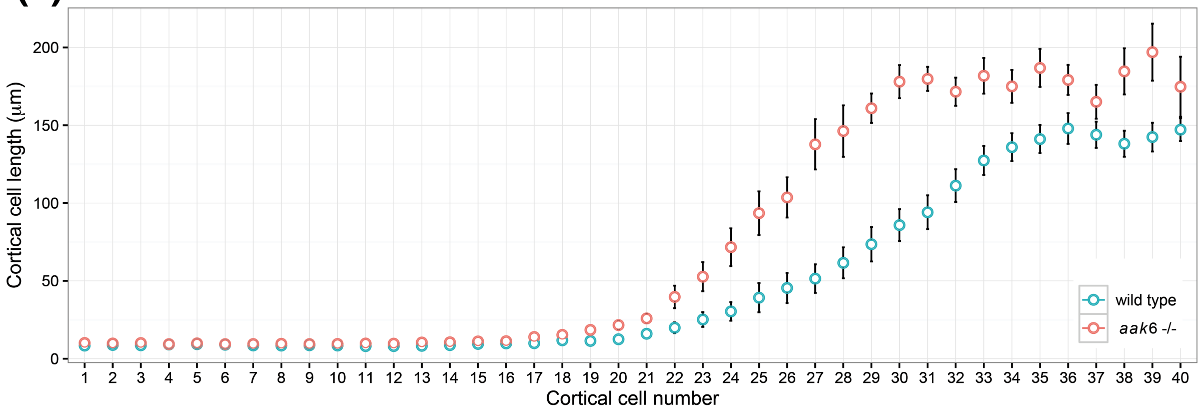

(b)

	Meristematic zone			Elongation zone			Mature zone		
	Zone length [µm]	Zone length [no. of cells]	Cell length average [µm]	Zone length [µm]	Zone length [no. of cells]	Cell length average [µm]	Distance from QC [µm]	Distance from QC [cells]	Cell length 5 cells average
**wild type**	199 ± 33	22 ± 3	9 ± 1	514 ± 94	10 ± 2	53 ± 12	713 ± 109	32 ± 3	150 ± 21
**aak6 -/-**	199 ± 60	19 ± 3	10 ± 2	546 ± 150	9 ± 3	66 ± 15	745 ± 164	27 ± 2	179 ± 27
**t-test p-value**	0.97	**0.03**	0.25	0.58	0.22	0.06	0.61	**0.01**	**0.02**

(c)

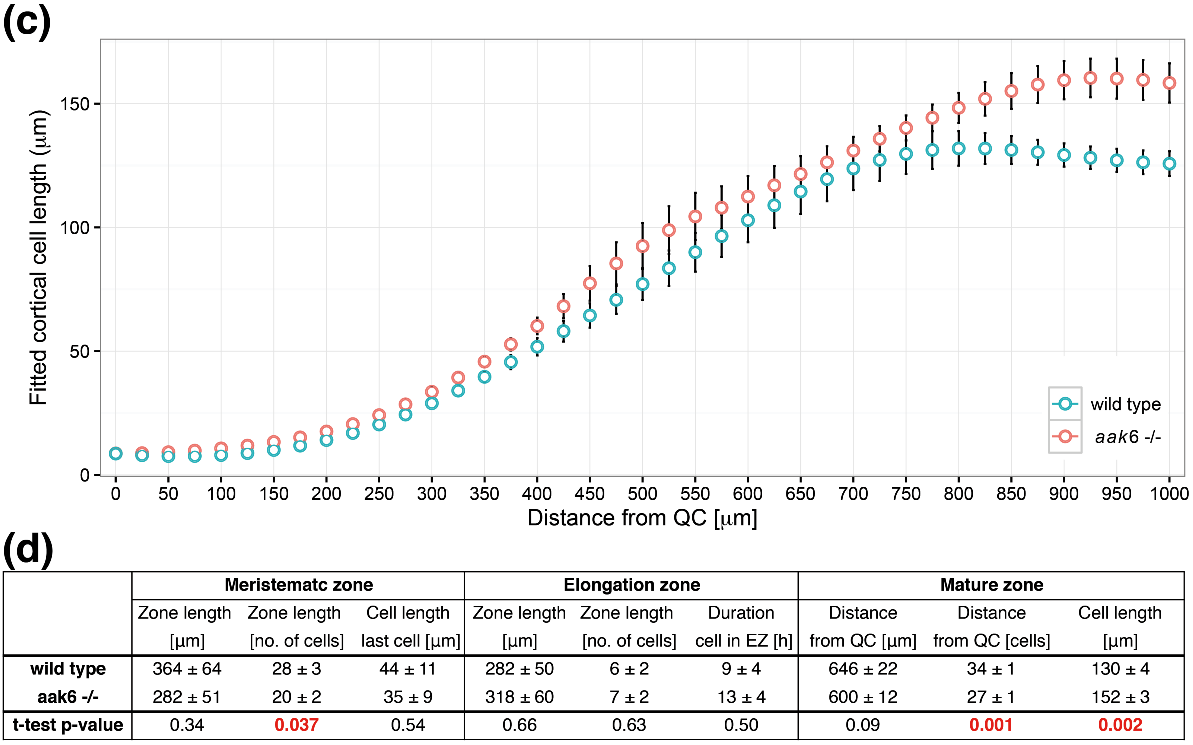

(d)

	Meristematc zone			Elongation zone			Mature zone		
	Zone length [µm]	Zone length [no. of cells]	Cell length last cell [µm]	Zone length [µm]	Zone length [no. of cells]	Duration cell in EZ [h]	Distance from QC [µm]	Distance from QC [cells]	Cell length [µm]
**wild type**	364 ± 64	28 ± 3	44 ± 11	282 ± 50	6 ± 2	9 ± 4	646 ± 22	34 ± 1	130 ± 4
**aak6 -/-**	282 ± 51	20 ± 2	35 ± 9	318 ± 60	7 ± 2	13 ± 4	600 ± 12	27 ± 1	152 ± 3
**t-test p-value**	0.34	**0.037**	0.54	0.66	0.63	0.50	0.09	**0.001**	**0.002**

Fig. S5 Root anatomy parameters of *aak6 -/-* compared to wild type. Root anatomy parameters evaluated from images taken by confocal microscope on fifth day after stratification: (a) Cortical cell length in relation to cellular distance from the quiescent center (distance in number of cells from QC); mean ± SE. (b) Cellular parameters for meristematic, elongation and mature zones; mean ± SD. P-values significant at alpha = 0.05 are highlighted in red. Root anatomy parameters evaluated from images taken by DIC microscopy on 8th day after stratification: (c) Fitted cortical cell length in relation to physical distance from the quiescent center; mean ± SE. (d) Cellular parameters for meristematic, elongation and mature zone; mean ± SE. P-values significant at alpha = 0.05 are highlighted in red.

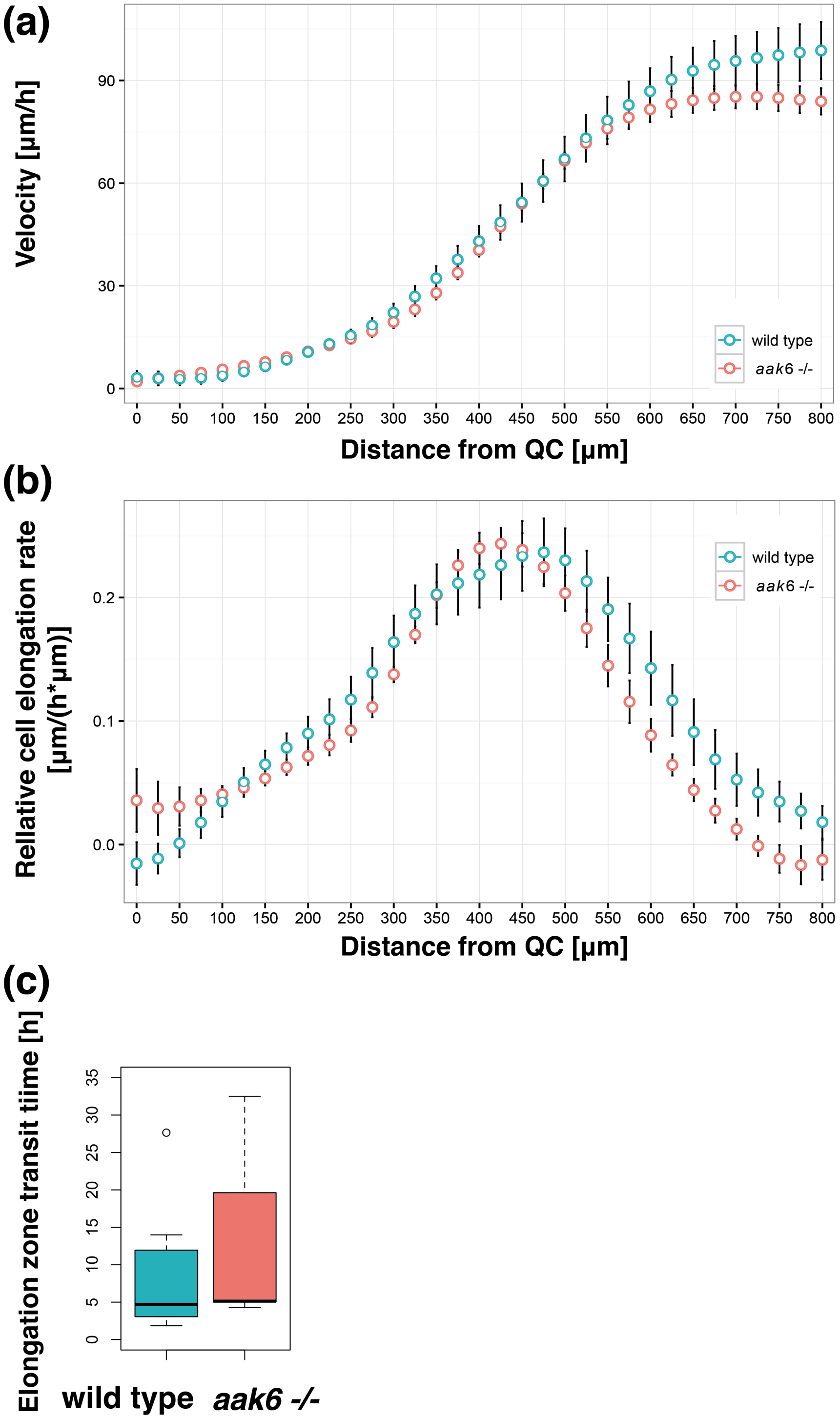

Fig. S6 Kinematic analysis of root growth. Kinematic analysis of root growth 8th day after stratification. (a) Root growth velocity profile of the wild type and *aak6 -/-* loss-of-function mutant; mean ± SE. (b) Relative cell elongation rate of wild type and *aak6 -/-* loss-of-function mutant; mean ± SE. (c) Time a cell spends in the elongation zone [h] in wild type and *aak6 -/-* loss-of-function mutant; mean values not significantly different (p-value = 0.50). Whiskers < 1.5 × IQR.

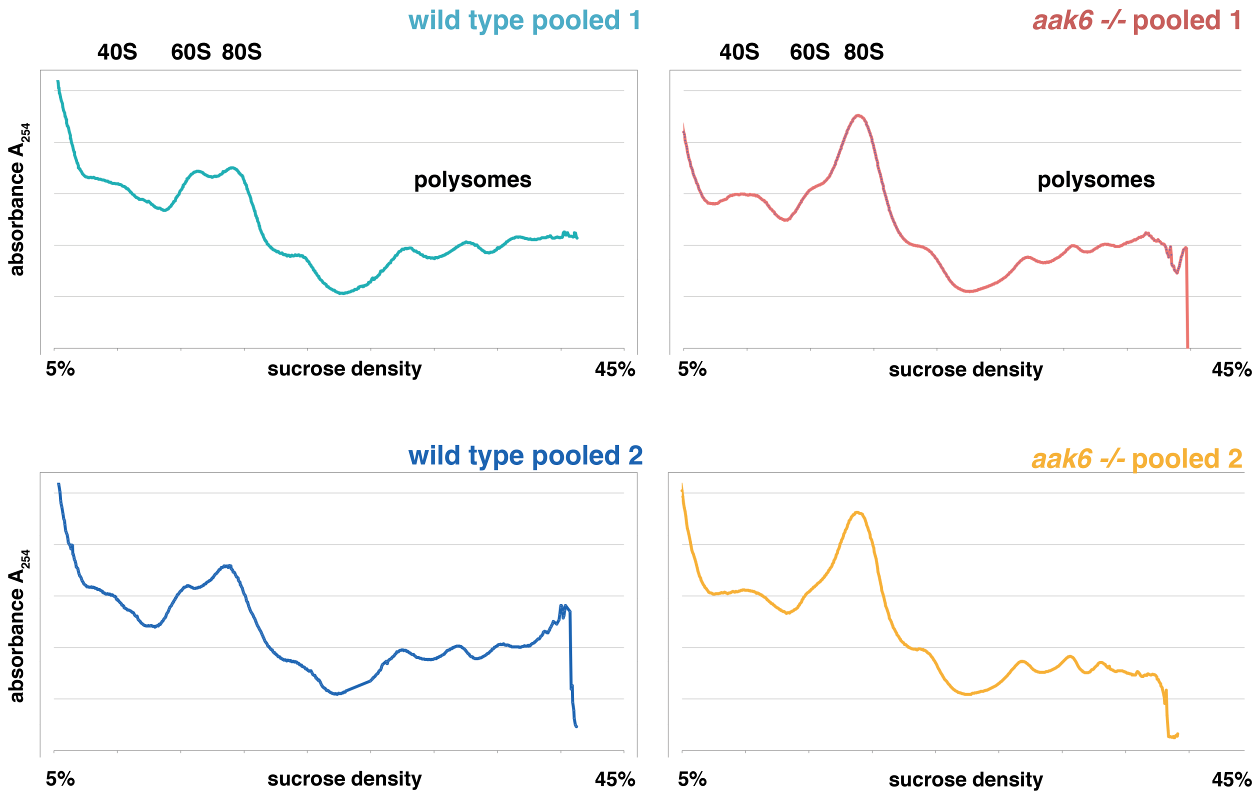

Fig. S7 Replicates of ribosome/polysome profiles. Spectrophotometric profiles of UV absorbance (254 nm) of sugar gradients. Ribosome/polysomal extracts were obtained from 3-week-old whole seedlings. Ribosomes were fractionated through 5-45% (w/V) sucrose gradient by 2.5 hour-long ultracentrifugation.

ACC_ID	Name	Origin
86	CUR-8	FRA
96	LAC-5	FRA
149	LDV-58	FRA
204	MIB-60	FRA
224	MIB-86	FRA
236	MOG-11	FRA
262	PAR-8	FRA
266	RAN	FRA
383	TOU-J-3	FRA
394	VOU-5	FRA
461	EM-183	UK
936	FOR-5	UK
1782	Ker-38	USA
1829	Mdn-1	USA
2057	Map-42	USA
2171	Paw-26	USA
2187	Pent-1	USA
2290	Ste-3	USA
2320	Wilcox-4	USA
5719	Bur-0	IRL
5723	Chr-1	UK
5729	Coc-1	ESP
5731	Crl-1	UK
5736	Ema-1	UK
5742	Frd-1	UK
5745	Hil-1	UK
5751	Kyl-1	UK
5752	Lan-1	UK
5837	Bor-1	CZE
6008	Duk	CZE
6016	Eds-1	SWE
6040	Kni-1	SWE
6064	Nyl-2	SWE
6074	Ör-1	SWE
6243	Tottarp-2	SWE
6730	CIBC-5	UK
6897	Ag-0	FRA
6898	An-1	BEL
6899	Bay-0	GER
6903	Bor-4	CZE
6904	Br-0	CZE
6906	C24	POR

ACC_ID	Name	Origin
6907	CIBC-17	UK
6909	Col-0	USA
6910	Ct-1	ITA
6911	Cvi-0	CPV
6913	Eden-2	SWE
6915	Ei-2	GER
6916	Est-1	RUS
6918	Fäb-4	SWE
6919	Ga-0	GER
6920	Got-22	GER
6921	Got-7	GER
6922	Gu-0	GER
6923	HR-10	UK
6924	HR-5	UK
6926	Kin-0	USA
6927	Kno-10	USA
6928	Kno-18	USA
6929	Kondara	TJK
6930	Kz-1	KZ
6931	Kz-9	KZ
6932	Ler-1	GER
6933	LL-0	ESP
6936	Lz-0	FRA
6937	Mrk-0	GER
6938	Ms-0	RUS
6939	Mt-0	LIB
6940	Mz-0	GER
6942	Nd-1	SUI
6943	NFA-10	UK
6944	NFA-8	UK
6945	Nok-3	NLD
6946	Oy-0	NOR
6951	Pu2-23	CZE
6956	Pu2-7	CZE
6958	Ra-0	FRA
6959	Ren-1	FRA
6960	Ren-11	FRA
6961	Se-0	ESP
6962	Sha	TJK
6963	Sorbo	TJK
6965	Spr1-6	SWE
6966	Sq-1	UK

ACC_ID	Name	Origin
6967	Sq-8	UK
6968	Tamm-2	FIN
6969	Tamm-27	FIN
6970	Ts-1	ESP
6971	Ts-5	ESP
6972	Tsu-1	JPN
6973	Ull2-3	SWE
6975	Uod-1	AUT
6976	Uod-7	AUT
6977	Van-0	CAN
6978	Wa-1	POL
6979	Wei-0	SUI
6980	Ws-0	RUS
6981	Ws-2	RUS
6982	Wt-5	GER
6983	Yo-0	USA
6984	Zdr-1	CZE
6985	Zdr-6	CZE
6987	Ak-1	GER
6990	Amel-1	NLD
6992	Ang-0	BEL
6994	Ann-1	FRA
6998	Arby-1	SWE
7000	Aa-0	GER
7002	Baa-1	NLD
7004	Bs-2	SUI
7008	7008	NL
7014	Ba-1	UK
7015	Bla-1	ESP
7026	Boot-1	UK
7028	Bch-1	GER
7031	Bsch-0	GER
7062	Ca-0	GER
7071	Chat-1	FRA
7075	Cit-0	FRA
7081	Co	POR
7092	Com-1	FRA
7094	Da-0	GER
7098	Di-1	FRA
7102	Do-0	GER
7123	Ep-0	GER
7126	Es-0	FIN

ACC_ID	Name	Origin
7143	Gel-1	NLD
7147	Gie-0	GER
7163	Ha-0	GER
7166	Hey-1	NLD
7169	Hh-0	GER
7172	Hl-3	GER
7176	Is-1	GER
7178	Jm-1	CZE
7181	Je-0	GER
7192	Kil-0	UK
7199	Kl-5	GER
7201	Kr-0	GER
7205	Krot-2	GER
7210	La-1	GER
7224	Li-3	GER
7231	Li-7	GER
7242	Lo-2	GER
7244	Mnz-0	GER
7246	Ma-2	GER
7252	Mc-0	UK
7255	Mh-0	POL
7262	Nw-4	GER
7268	Np-0	GER
7275	No-0	GER
7276	Ob-0	GER
7280	Old-1	GER
7283	Ors-1	ROM
7287	Ove-0	GER
7291	Pa-2	ITA
7297	Pf-0	GER
7299	Pi-2	AUT
7300	Pla-0	ESP
7306	Pog-0	CAN
7307	Pn-0	FRA
7309	Po-1	GER
7310	Pr-0	GER
7316	Rhen-1	NLD
7317	Ri-0	CAN
7320	Rou-0	FRA
7330	Sapporo-0	JPN
7331	Sh-0	GER
7337	Si-0	GER

ACC_ID	Name	Origin
7344	Sg-1	GER
7351	Ty-0	UK
7352	Te-0	FIN
7353	Tha-1	NLD
7354	Ting-1	SWE
7355	Tiv-1	ITA
7372	Tscha-1	AUT
7378	Uk-1	GER
7382	Utrecht	NLD
7384	Ven-1	NLD
7404	Wc-1	GER
7418	Zu-1	SUI
7424	Jl-3	CZE
7438	N13	RUS
7477	WAR	USA
7514	RRS-7	USA
7515	RRS-10	USA
7516	Vår2-1	SWE
7519	ÖMö2-3	SWE
7520	Lp2-2	CZE
7521	Lp2-6	CZE
7522	Mr-0	ITA
7523	Pna-17	USA
7524	Rmx-A02	USA
7525	Rmx-A180	USA
7526	Pna-10	USA
8213	Pro-0	ESP
8214	Gy-0	FRA
8215	Fei-0	POR
8230	Algutsrum	SWE
8231	Brö1-6	SWE
8233	Dem-4	USA
8236	HSm	CZE
8239	Köln	GER
8240	Kulturen-1	SWE
8241	Liarum	SWE
8243	PHW-2	ITA
8245	Seattle-0	USA
8247	San-2	SWE
8249	Vimmerby	SWE
8256	Bå1-2	SWE
8258	Bå4-1	SWE
8259	Bå5-1	SWE
8265	Blh-1	CZE
8270	Bs-1	SUI
8271	Bu-0	GER
8274	Can-0	ESP
8284	DraII-1	CZE
8285	DraIII-1	CZE
8290	En-1	GER
8296	Gd-1	GER
8297	Ge-0	SUI
8300	Gr-1	AUT
8304	Hi-0	NLD
8306	Hov4-1	SWE
8310	Hs-0	GER
8311	In-0	AUT
8312	Is-0	GER
8313	Jm-0	CZE
8314	Ka-0	AUT
8325	Lip-0	POL
8329	Lm-2	FRA
8334	Lu-1	SWE
8343	Na-1	FRA
8348	Nw-0	GER
8351	Ost-0	SWE
8353	Pa-1	ITA
8354	Per-1	RUS
8365	Rak-2	CZE
8366	Rd-0	GER
8369	Rev-1	SWE
8374	Rsch-4	RUS
8376	Sanna-2	SWE
8378	Sap-0	CZE
8387	St-0	SWE
8388	Stw-0	RUS
8395	Tu-0	ITA
8420	Kelsterbach-4	GER
9104	Lag1-6	GEO
9152	Cala-8	ESP
9165	Truk-5	UKR
9302	Edinburgh-5	UK
9308	Ullapool-3	UK
100000	Wil-1-Dean-Lab	LTU

Table S1 252 *Arabidopsis thaliana* accessions used in this study.

Seed batch	SALK ID	Position	Target gene	T-DNA presence	Primary root length (Total root length trait)					Root growth rate					Reative root growth rate				
					DAS3	DAS4	DAS 5	DAS 6	DAS 7	DAS 3-4	DAS 4-5	DAS 5-6	DAS 6-7	DAS 3-7 average	DAS 3-4	DAS 4-5	DAS 5-6	DAS 6-7	DAS 3-7 average
L0901	SALK_134312	UTR5	AT2G22905	homozygous	0.560	0.834	0.714	0.724	0.766	0.875	0.952	0.517	0.995	0.634	0.856	0.555	0.455	0.943	0.356
L0905	SALK_134312	UTR5	AT2G22905	homozygous	0.059	0.319	0.546	0.935	0.692	0.732	0.535	0.638	0.437	0.207	0.109	0.191	0.540	0.286	0.396
L1001	SALK_131000	UTR3/promotor	AT2G22905/AT2G22910	homozygous	0.047	0.005	0.001	0.000	0.004	0.012	0.042	0.074	0.285	0.004	0.241	0.229	0.619	0.283	0.701
L1007	SALK_131000	UTR3/promotor	AT2G22905/AT2G22910	homozygous	0.007	0.012	0.092	0.048	0.169	0.641	0.817	0.480	0.303	0.182	0.028	0.061	0.292	0.527	0.295
L1103	SALK_124797	UTR3/promotor	AT2G22905/AT2G22910	homozygous	0.028	0.016	0.073	0.048	0.176	0.494	0.556	0.613	0.934	0.560	0.026	0.017	0.094	0.338	0.065
L1106	SALK_124797	UTR3/promotor	AT2G22905/AT2G22910	homozygous	0.000	0.003	0.002	0.006	0.016	0.213	0.624	0.851	0.742	0.378	0.018	0.014	0.015	0.135	0.024
L1304	SALK_004190	UTR5	AT2G22920	homozygous	0.063	0.496	0.160	0.809	0.571	0.744	0.685	0.611	0.781	0.646	0.019	0.411	0.413	0.306	0.442
L1310	SALK_004190	UTR5	AT2G22920	homozygous	0.240	0.039	0.894	0.056	0.060	0.201	0.667	0.045	0.293	0.075	0.750	0.449	0.141	0.504	0.330
L2202	SALK_015289	UTR5	AT5G60340	homozygous	0.000	0.000	0.000	0.002	NA	0.015	0.684	0.770	NA	0.598	0.005	0.004	0.002	NA	0.031
L2203	SALK_015289	UTR5	AT5G60340	homozygous	0.000	0.000	0.000	0.000	0.000	0.030	0.937	0.692	0.012	0.088	0.001	0.001	0.005	0.019	0.005
L2303	SALK_061336	promotor/promotor	AT5G60340/AT5G60350	homozygous	0.536	0.927	0.283	0.885	0.347	0.486	0.104	0.515	0.622	0.492	0.355	0.156	0.385	0.720	0.468
L2305	SALK_061336	promotor/promotor	AT5G60340/AT5G60350	homozygous	0.257	0.683	0.396	0.209	0.244	0.025	0.145	0.029	0.529	0.017	0.016	0.438	0.110	0.855	0.145
L2401	SALK_088228	UTR5	AT5G60350	homozygous	0.057	0.011	0.357	0.009	0.102	0.040	0.386	0.465	0.128	0.214	0.161	0.096	0.358	0.473	0.891
L2402	SALK_088228	UTR5	AT5G60350	homozygous	0.434	0.159	0.200	0.131	0.165	0.158	0.970	0.468	0.351	0.675	0.654	0.180	0.036	0.966	0.677

Table S2 Phenotypes of T-DNA insertion mutant lines (t-test p-values). Bonferroni corrected significance threshold for multiple testing: alpha = 0.0033 (15 t-tests). P-values significant at this corrected threshold are highlighted in red.

ID	code	target	sequence (5'-3')
P1	LBa1	T-DNA	TGGTTCACGTAGTGGGCCATCG
P2	SALK_134312_left	AT2G22905	AGATTCCATTACGCACACCTG
P3	SALK_134312_right	AT2G22905	CTCCGTTGTCGAAGAAGACAC
P4	SALK_131000_left	AT2G22905, AT2G22910	TTTTGTTTGGAATGCGTTTTC
P5	SALK_131000_right	AT2G22905, AT2G22910	ACACTCGTGAACCGGTATTTG
P6	SALK_124797_left	AT2G22910	GTGTCTTCTTCGACAACGGAG
P7	SALK_124797_right	AT2G22910	AAACACCTTGCGTGCATAAAC
P8	SALK_004190_left	AT2G22920	TTCCAACGCTATAGTGGCATC
P9	SALK_004190_right	AT2G22920	GGTTTCGAGTTCGAAAGGAAG
P10	SALK_015289_left	AT5G60340	CCCTATGGCAAATCACAAATG
P11	SALK_015289_right	AT5G60340	GTCACAGCCATGGTAGTCCAC
P12	SALK_061336_left	AT5G60340, AT5G60350	CTACCTTGGTTTTTGCTGCTG
P13	SALK_061336_right	AT5G60340, AT5G60350	CGGTAGTGAACAAACAAACACC
P14	SALK_088228_left	AT5G60350	TTTGTGATTTGCCATAGGGAG
P15	SALK_088228_right	AT5G60350	TTGTTGTCTTCCACCAAAAGG
P16	35S_Infusion_FW	35S, pGreen0299	CGGTATCGATAAGCTTAACATGGTGGAGCACGAC
P17	35S_Infusion_RV	pGreen0229, 35S	CTGCAGGAATTCGATATCCTATCGTTCGTAAATGGTGA
P18	Infusion_FW_AAK6_pGreen	35S, AT5G60340	TACGAACGATAGGATGAAGACCGAACATGGCGAGA
P19	Infusion_RW_AAK6_pGreen	pGreen0229, AT5G60340	GACGTCCTTAAGCTACTAGTAGTCCCAACGGTACG
P20	Infusion_gAAK6_forward	AT5G60340, pGreen0229	CGGTATCGATAAGCTTGTGATGTCCCCCATTGTGGT
P21	Infusion_gAAK6_reverse	AT5G60340, pGreen0229	CTGCAGGAATTCGATATCGCTTGAAGGATGTGTATGATCCAG
P22	ATTB4_AAK6	AT5G60340	GGGGACAACTTTGTATAGAAAAGTTGCGGTGATGTCCCCCATTGT
P23	ATTB1rev_AAK6	AT5G60340	GGGGACTGCTTTTTTGTACAAACTTGCttctatgaagcctgaagaaatt
P24	ATTB1_AAK6	AT5G60340	GGGGACAAGTTTGTACAAAAAAGCAGGCTTCAGAAGACCGAACATGGCGA
P25	ATTB2_AAK6	AT5G60340	GGGGACCACTTTGTACAAGAAAGCTGGGTTGGGTTGCCATGCATTAATCC
P26	Tubulin2_Exon1_forward	AT5G62690	GGCGCCAAGTTCTGGGAAGTGG
P27	Tubulin2_Exon1_reverse	AT5G62690	CCATGAGCACTGCACGAGGAACG
P28	AAK6_Exon2_forward	AT5G60340	TGCTATGATAGAAGGAGGGAACA
P29	AAK6_Exon2_reverse	AT5G60340	TAAACGGTCATACAAAACGGAGT

Table S3 Primers used in this study.

Trait	standard deviation median SD **within** accessions		standard deviation [mm] between accessions		heritability		Pearson's r $trait_{random}$ vs. $trait_{controlled}$	Pearson's r accession's seed area vs.	
	random seeds	controlled seeds	random seeds	controlled seeds	random seeds	controlled seeds		$trait_{random}$	$trait_{controlled}$
Total_length_DAS3 [mm]	1.0	0.9	1.1	0.8	**0.44**	0.43	0.71	0.20	0.03
Total_length_DAS4 [mm]	1.5	1.1	1.6	1.3	**0.50**	0.50	0.76	0.33	0.06
Total_length_DAS5 [mm]	1.8	1.3	2.0	1.6	0.49	**0.51**	0.74	0.39	0.08
Total_length_DAS6 [mm]	2.2	1.5	2.5	1.9	0.50	**0.51**	0.74	0.43	0.11
Total_length_DAS7 [mm]	2.6	1.8	2.9	2.3	0.49	**0.50**	0.75	0.43	0.14
Euclidian_length_DAS3 [mm]	0.9	0.8	1.0	0.8	**0.45**	0.43	0.72	0.21	0.03
Euclidian_length_DAS4 [mm]	1.4	1.0	1.5	1.2	**0.51**	0.49	0.75	0.33	0.07
Euclidian_length_DAS5 [mm]	1.7	1.2	1.9	1.5	0.49	**0.51**	0.74	0.39	0.09
Euclidian_length_DAS6 [mm]	2.0	1.4	2.3	1.8	0.50	**0.50**	0.73	0.43	0.11
Euclidian_length_DAS7 [mm]	2.4	1.7	2.7	2.2	0.49	**0.50**	0.74	0.43	0.14
Root_tortuosity_DAS3	0.04	0.04	0.0	0.0	**0.08**	0.07	0.21	-0.10	-0.02
Root_tortuosity_DAS4	0.05	0.04	0.1	0.0	0.03	**0.06**	0.14	-0.02	0.05
Root_tortuosity_DAS5	0.04	0.03	0.0	0.0	0.04	**0.08**	0.34	0.00	0.00
Root_tortuosity_DAS6	0.04	0.03	0.0	0.0	0.04	**0.09**	0.32	-0.03	-0.02
Root_tortuosity_DAS7	0.03	0.03	0.0	0.0	0.08	**0.12**	0.48	-0.08	-0.04
Root_tortuosity_avg	0.04	0.03	0.0	0.0	0.03	**0.09**	0.29	-0.05	0.00
Root_growth_rate_DAS3-DAS4 [mm]	0.6	0.4	0.7	0.5	**0.50**	0.48	0.73	0.44	0.21
Root_growth_rate_DAS4-DAS5 [mm]	0.5	0.4	0.6	0.4	0.44	**0.44**	0.70	0.41	0.16
Root_growth_rate_DAS5-DAS6 [mm]	0.5	0.4	0.6	0.5	**0.47**	0.46	0.77	0.43	0.21
Root_growth_rate_DAS6-DAS7 [mm]	0.6	0.5	0.6	0.5	**0.44**	0.41	0.70	0.30	0.18
Root_growth_rate_avg [mm]	0.5	0.4	0.6	0.4	0.45	**0.50**	0.76	0.44	0.21
Relative_root_growth_rate_DAS3-DAS4	0.4	0.5	1.2	1.3	0.12	**0.23**	0.60	0.03	0.01
Relative_root_growth_rate_DAS4-DAS5	0.1	0.1	0.3	0.4	**0.22**	0.15	0.60	-0.08	0.12
Relative_root_growth_rate_DAS5-DAS6	0.1	0.1	0.1	0.1	0.13	**0.19**	0.80	0.02	0.08
Relative_root_growth_rate_DAS6-DAS7	0.0	0.0	0.1	0.1	0.18	**0.26**	0.56	-0.18	0.11
Relative_root_growth_rate_avg	0.2	0.2	0.3	0.3	0.13	**0.18**	0.69	-0.03	0.04
Root_angle_DAS3	12.0	11.0	5.1	3.7	0.01	**0.01**	-0.09	0.07	-0.02
Root_angle_DAS4	9.2	8.4	4.7	4.2	0.04	**0.07**	0.33	-0.01	-0.06
Root_angle_DAS5	7.2	6.7	4.8	4.3	0.08	**0.18**	0.56	-0.05	-0.07
Root_angle_DAS6	5.9	5.7	4.2	4.5	0.18	**0.28**	0.73	-0.05	-0.03
Root_angle_DAS7	5.4	5.1	4.2	4.7	0.25	**0.35**	0.75	-0.03	-0.03
Root_angle_avg	7.6	6.9	4.1	3.8	0.06	**0.14**	0.48	0.03	-0.04
Root_direction_index_DAS3	0.14	0.13	0.1	0.1	0.08	**0.09**	0.37	-0.07	0.04
Root_direction_index_DAS4	0.11	0.11	0.1	0.1	**0.15**	0.12	0.50	-0.05	-0.05
Root_direction_index_DAS5	0.09	0.08	0.1	0.1	**0.22**	0.20	0.62	-0.01	-0.07
Root_direction_index_DAS6	0.08	0.07	0.1	0.1	**0.31**	0.25	0.61	-0.08	-0.05
Root_direction_index_DAS7	0.07	0.07	0.1	0.1	**0.35**	0.28	0.59	-0.12	-0.07
Root_direction_index_avg	0.09	0.08	0.1	0.1	**0.20**	0.17	0.56	-0.08	-0.05
Root_horizontal_index_DAS3 [mm]	0.1	0.1	0.1	0.1	0.13	**0.16**	0.64	0.19	0.05
Root_horizontal_index_DAS4 [mm]	0.2	0.1	0.1	0.1	**0.16**	0.15	0.67	0.26	-0.04
Root_horizontal_index_DAS5 [mm]	0.2	0.2	0.1	0.1	**0.24**	0.22	0.75	0.23	-0.03
Root_horizontal_index_DAS6 [mm]	0.2	0.2	0.2	0.2	**0.34**	0.32	0.83	0.21	0.01
Root_horizontal_index_DAS7 [mm]	0.3	0.2	0.3	0.2	**0.40**	0.39	0.84	0.19	0.01
Root_horizontal_index_avg [mm]	0.2	0.1	0.1	0.1	0.26	**0.27**	0.77	0.26	0.00
Root_vertical_index_DAS3 [mm]	0.3	0.2	0.3	0.2	**0.44**	0.41	0.71	0.20	0.03
Root_vertical_index_DAS4 [mm]	0.4	0.3	0.4	0.3	**0.49**	0.49	0.75	0.33	0.07
Root_vertical_index_DAS5 [mm]	0.5	0.4	0.5	0.4	0.48	**0.51**	0.74	0.39	0.09
Root_vertical_index_DAS6 [mm]	0.6	0.4	0.7	0.5	0.49	**0.50**	0.73	0.42	0.11
Root_vertical_index_DAS7 [mm]	0.7	0.5	0.8	0.6	0.49	**0.50**	0.75	0.43	0.15
Root_vertical_index_avg [mm]	0.7	0.5	0.5	0.4	0.33	**0.35**	0.70	0.41	0.11
Root_linearity_DAS3	0.32	0.32	0.1	0.1	0.02	**0.02**	0.03	0.18	0.05
Root_linearity_DAS4	0.32	0.32	0.1	0.1	0.02	**0.03**	0.11	0.10	-0.03
Root_linearity_DAS5	0.32	0.31	0.1	0.1	0.04	**0.05**	0.24	-0.02	-0.02
Root_linearity_DAS6	0.30	0.31	0.1	0.1	0.09	**0.09**	0.44	0.04	0.07
Root_linearity_DAS7	0.30	0.30	0.1	0.1	0.13	**0.15**	0.54	0.03	0.06
Root_linearity_avg	0.22	0.21	0.1	0.1	0.06	**0.09**	0.30	0.11	0.03
Average_root_width_DAS3 [mm]	0.014	0.013	0.0	0.0	**0.13**	0.09	0.41	0.27	0.05
Average_root_width_DAS4 [mm]	0.011	0.009	0.0	0.0	**0.17**	0.14	0.59	0.37	0.19
Average_root_width_DAS5 [mm]	0.009	0.007	0.0	0.0	0.16	**0.17**	0.63	0.44	0.19
Average_root_width_DAS6 [mm]	0.010	0.007	0.0	0.0	0.23	**0.44**	-0.03	0.26	0.12
Average_root_width_DAS7 [mm]	0.009	0.007	0.0	0.0	0.24	**0.41**	0.70	0.25	0.15
Average_root_width_avg [mm]	0.010	0.008	0.0	0.0	0.16	**0.20**	0.57	0.39	0.18
Root_width_20_DAS3 [mm]	0.035	0.035	0.0	0.0	**0.08**	0.05	0.29	0.02	-0.13
Root_width_20_DAS4 [mm]	0.026	0.027	0.0	0.0	**0.11**	0.08	0.38	0.11	-0.08
Root_width_20_DAS5 [mm]	0.021	0.021	0.0	0.0	**0.11**	0.11	0.56	0.09	-0.01
Root_width_20_DAS6 [mm]	0.018	0.018	0.0	0.0	0.13	**0.21**	0.42	0.09	0.00
Root_width_20_DAS7 [mm]	0.017	0.017	0.0	0.0	0.20	**0.24**	0.76	0.07	-0.03
Root_width_20_avg [mm]	0.022	0.022	0.0	0.0	**0.11**	0.11	0.60	0.10	-0.03
Root_width_40_DAS3 [mm]	0.021	0.022	0.0	0.0	**0.12**	0.07	0.46	0.12	-0.02
Root_width_40_DAS4 [mm]	0.013	0.013	0.0	0.0	0.15	**0.16**	0.65	0.30	0.19
Root_width_40_DAS5 [mm]	0.011	0.008	0.0	0.0	**0.17**	0.17	0.69	0.41	0.18
Root_width_40_DAS6 [mm]	0.011	0.008	0.0	0.0	0.18	**0.39**	0.06	0.32	0.15
Root_width_40_DAS7 [mm]	0.010	0.007	0.0	0.0	0.20	**0.34**	0.66	0.34	0.17
Root_width_40_avg [mm]	0.011	0.010	0.0	0.0	0.16	**0.17**	0.60	0.35	0.17
Root_width_60_DAS3 [mm]	0.013	0.013	0.0	0.0	**0.16**	0.10	0.55	0.31	0.12
Root_width_60_DAS4 [mm]	0.011	0.009	0.0	0.0	0.17	**0.17**	0.69	0.39	0.27
Root_width_60_DAS5 [mm]	0.010	0.007	0.0	0.0	**0.18**	0.16	0.59	0.47	0.25
Root_width_60_DAS6 [mm]	0.010	0.007	0.0	0.0	0.23	**0.39**	-0.08	0.30	0.16
Root_width_60_DAS7 [mm]	0.010	0.007	0.0	0.0	0.19	**0.36**	0.61	0.32	0.19
Root_width_60_avg [mm]	0.009	0.007	0.0	0.0	0.18	**0.23**	0.51	0.44	0.23
Root_width_80_DAS3 [mm]	0.012	0.010	0.0	0.0	**0.14**	0.08	0.49	0.45	0.27
Root_width_80_DAS4 [mm]	0.011	0.008	0.0	0.0	**0.17**	0.14	0.55	0.41	0.32
Root_width_80_DAS5 [mm]	0.010	0.007	0.0	0.0	0.17	**0.18**	0.53	0.48	0.25
Root_width_80_DAS6 [mm]	0.010	0.008	0.0	0.0	0.24	**0.41**	-0.14	0.25	0.16
Root_width_80_DAS7 [mm]	0.010	0.007	0.0	0.0	0.20	**0.36**	0.53	0.27	0.21
Root_width_80_avg [mm]	0.009	0.006	0.0	0.0	0.18	**0.23**	0.41	0.46	0.27
Root_width_100_DAS3 [mm]	0.012	0.009	0.0	0.0	0.12	**0.13**	0.40	0.50	0.31
Root_width_100_DAS4 [mm]	0.011	0.008	0.0	0.0	0.13	**0.13**	0.51	0.45	0.30
Root_width_100_DAS5 [mm]	0.010	0.007	0.0	0.0	0.15	**0.16**	0.57	0.48	0.24
Root_width_100_DAS6 [mm]	0.010	0.008	0.0	0.0	0.28	**0.39**	-0.11	0.20	0.13
Root_width_100_DAS7 [mm]	0.010	0.007	0.0	0.0	0.19	**0.37**	0.48	0.20	0.21
Root_width_100_avg [mm]	0.009	0.006	0.0	0.0	0.15	**0.24**	0.40	0.46	0.28

Table S4 Impact of seed size on root growth traits. Heritability and correlation of seed area with early root growth traits are compared within a column and assigned shading on a two-color scale (white - green) with higher values highlighted in green.

```
slow  Bå4-1    1  MARRNRGGTRRARPNLLITGTPGTGKSTTAAALAEATNLRYICIGDLVKEKEFYHGWDSE   60
fast |Col-0    1  MARRNRGVTRRERPNLLITGTPGTGKSTTASALAEATNLRYICIGDLVKEKEFYHGWDNE   60
     |Tha-1    1  MARRNRGVTRRERPNLLITGTPGTGKSTTASALAEATNLRYICIGDLVKEKEFYHGWDNE   60
                  ******* *** ******************:***************************.*

slow  Bå4-1   61  LECHFINEDSVIDELDDAMIEGGNIVDYHGCDFFPQRWFDRVVVLRTENSVLYDRLTNRG  120
fast |Col-0   61  LECHFINEDSVIDELDDAMIEGGNIVDYHGCDFFPQRWFDRVVVLRTENSVLYDRLTNRG  120
     |Tha-1   61  LECHFINEDSVIDELDDAMIEGGNIVDYHGCDFFPQRWFDRVVVLRTENSVLYDRLTNRG  120
                  ************************************************************

slow  Bå4-1  121  YSGTKLSNNLQCEMYQVLLEEAHDSYDEEIVTELQSNTIEDISNNVSTLTDWINAWQP    178
fast |Col-0  121  YSGTKLSNNLQCEMYQVLLEEAHDSYDEEIVTELQSNTIEDISNNVSTLTDWINAWQP    178
     |Tha-1  121  YSGTKLSNNLQCEMYQVLLEEAHDSYDEEIVTELQSNTIEDISNNVSTLTDWINAWQP    178
                  **********************************************************
```

Table S6 Alignment of *AAK6* coding sequences (amino acids) of Bå4-1, Col-0, and Tha-1.

	Col-0 pooled root tips S1	Col-0 pooled root tips S2	Col-0 pooled root tips S3	Col-0 pooled root tips S4	Col-0 pooled root tips S5	Col-0 pooled root tips S6	Average	Average ploidy class proportion
16C	1029	966	913	912	937	951	951	11%
8C	2633	2795	2676	2866	2987	2821	2796	33%
4C	3028	2804	2943	2948	2911	2940	2929	34%
2C	1825	1824	1908	1763	1962	1896	1863	22%

	aak6 pooled root tips S1	aak6 pooled root tips S2	aak6 pooled root tips S3	aak6 pooled root tips S4	aak6 pooled root tips S5	aak6 pooled root tips S6	Average	Average ploidy class proportion
16C	1820	1362	1574	1617	1534	1573	1580	18%
8C	2616	3047	2593	2964	2850	3007	2846	33%
4C	2542	2580	2563	2481	2790	2617	2596	30%
2C	1560	1680	1729	1667	1694	1610	1657	19%

Difference in proportions of ploidy classes in Col-0 vs. *aak6 -/-*, t-test p-values:		
16C	0.000	***
8C	0.617	
4C	0.000	***
2C	0.000	***

Table S7 Proportions of nuclear ploidy classes in the root apices. Counts of nuclei in discrete nuclear ploidy classes, fifth day after stratification. Bonferroni corrected significance threshold for multiple testing: alpha = 0.0125 (4 t-tests). P-values significant at this corrected threshold are highlighted in red as well as by "***".

Methods S1

Relative expression fold change of the *AAK6* gene

We tested the expression of *AAK6* gene (AT5G60340) in Col-0, *aak6* mutant, and *35S:AAK6* line; *Tubulin 2* (AT5G62690) was used as the reference gene (used primers P26 – P29 are listed in Supplemental Table S3).

Seeds were surface sterilized, stratified and cultured on 1x MS, 1% sucrose, 0.8% agar plates. Seedlings grew until 5th day after stratification. 5 mm root tips were collected, frozen in liquid nitrogen and ground in 1.5 ml Eppendorf tubes using glass beads and Retsch MM 400 mill. Total RNA was extracted by Maxwell 16 LEV simplyRNA Tissue Kit (Promega) including DNAse treatment according to manufacturer's instructions. cDNA was generated by two-step RT-PCR: 200 ng of DNAse treated RNA and 0.5 µg of Oligo(dT) primer were added on ice to each PCR-tube to a total of 12.5 l DEPC-treated water solution and then incubated at 65 °C, 5 min. RT master mix was prepared from: 2 l of 10 mM dNTP mix, 0.5 l of RiboLock RNase inhibitor, 1 l of RevertAid Reverse Transcriptase (RAT) and 4 l of 5x RAT reaction buffer (all ThermoFisher Scientific) for each sample. 7.5 l of RT master mix was added to template RNA and Oligo(dT) primer. The following RT-PCR program was run in Tetrad 2 PCR thermal cycler (Biorad): 10 min 25 °C, 60 min 42 °C, 10 min 70 °C. The resulting cDNA was analysed immediately by a real-time qPCR. qPCR master mix was prepared from: 12.5 l 2x SensiMix SYBR & Fluorescein Kit (PEQLAB), 0.63 l of 10 M forward and reverse primers for *Tubulin 2* and *AAK6* each. 2.5 l of 2x diluted template cDNA was added to 22.5 l of qPCR master mix in wells on 96-well plate. qPCR was performed on Roche LightCycler 96 as follows: 10 min 95 °C, 40 x (15 s 95 °C, 15 s 58 °C, 15 s 72 °C), 10 s 95 °C, 60 s 65 °C, and 1s 97 °C. Relative fold-change was evaluated by the delta-delta Ct method using 4 pooled biological by 3 technical replicates for each genotype.

Methods S2

Seed size measurements

To assess the correlation of average seed size and BRAT evaluated root growth traits, we measured 2D seed area [mm^2] from 1600-dpi images scanned by Perfection V600 scanner (Epson). Acquired images were downscaled to 8-bit, threshold (20, 255) and converted to binary mask. Binary images were then analyzed in Fiji using plugin 'Analyze Particles' (inclusion parameters: size = 0.04-0.23 mm^2 , circularity = 0.80-1.00).

Average seed sizes were evaluated for 252 accessions (n > 500 for each).

Notes S1

Precautions taken in propagation of natural accessions

To avoid unnecessary maternal effects, we only used seeds harvested from *Arabidopsis thaliana* plants grown under the same conditions in the same growth chamber at the same time.

Notes S2

Precautions taken with SALK T-DNA lines

To avoid unnecessary maternal effects, T-DNA plants were propagated side-by-side under the same growth conditions as the control accession Col-0 (Columbia-0). We confirmed the homozygosity of the T-DNA insertion by genotyping (primers are listed in the Supplemental Table S3), cultured and phenotyped the F1 generation of homozygous seedlings side-by-side with the respective control accession Col-0.

Notes S3

Increase in endopolyploidy levels accompanies increase in mature cell size in *aak6* mutant

We report larger mature cells and increased endopolyploidy levels in the *aak6* roots. This raises a question whether the additional endocycles are important for post-mitotic compensatory cell enlargement in the root or in other plant organs in general. Increased endopolyploidy levels have long been associated with increased cell sizes (reviewed in (Sugimoto-Shirasu and Roberts, 2003)). While it has been reported that endopolyploidization can precede rapid cell elongation during normal root development (Hayashi *et al.*, 2013), several lines of evidence suggest that endopolyploidization is, at least in some cases, not required for the compensatory cell enlargement. To list some examples, compensatory cell enlargement takes place in the absence of enhanced endoreplication in the strong overexpressor of Kip-related protein 2 (KRP2; (De Veylder *et al.*, 2001)), in the 2C guard cells (Autran *et al.*, 2002) or in the *an3 xs6* double mutant (Fujikura *et al.*, 2007). And finally, lack of CTD1, the DNA replication licensing factor, causes defects in cell division, increased endopolyploidy levels but smaller cells (Raynaud *et al.*, 2005), further illustrating that increases in ploidy levels are not necessarily required for compensatory cell enlargement. Taken together, while compensatory cell enlargement doesn't necessarily involve increased endopolyploidy levels, the compensatory effects that we observe in the *aak6* mutant are accompanied by it. However, it remains to be untangled to what degree does the post-mitotic cellular growth depend on the DNA content in either normal or compensatory root cell enlargement.

Autran D, Jonak C, Belcram K, Beemster GTS, Kronenberger J, Grandjean O, Inze D and Traas J. 2002. Cell numbers and leaf development in Arabidopsis: a functional analysis of the STRUWWELPETER gene. *The EMBO journal* **21:** 6036–6049.

De Veylder L, Beeckman T, Beemster GTS, Krols L, Terras F, Landrieu I, Van Der Schueren E, Maes S, Naudts M and Inzé D. 2001. Functional Analysis of Cyclin-Dependent Kinase Inhibitors of Arabidopsis. *The Plant Cell* **13:** 1653 LP – 1668.

Fujikura U, Horiguchi G and Tsukaya H. 2007. Genetic relationship between angustifolia3 and extra-small sisters highlights novel mechanisms controlling leaf size. *Plant signaling & behavior* **2:** 378–380.

Hayashi K, Hasegawa J and Matsunaga S. 2013. The boundary of the meristematic and elongation zones in roots: endoreduplication precedes rapid cell expansion. *Scientific reports* **3:** 2723.

Raynaud C, Perennes C, Reuzeau C, Catrice O, Brown S and Bergounioux C. 2005. Cell and plastid division are coordinated through the prereplication factor AtCDT1. *Proceedings of the National Academy of Sciences* **102:** 8216–8221.

Sugimoto-Shirasu K and Roberts K. 2003. "Big it up": endoreduplication and cell-size control in plants. *Current Opinion in Plant Biology* **6:** 544–553.
